# Short-Term Effects of Human versus Bovine Sialylated Milk Oligosaccharide Microinjection on Zebrafish Larvae Survival, Locomotor Behavior and Gene Expression

**DOI:** 10.3390/ijms24065456

**Published:** 2023-03-13

**Authors:** Rosario Licitra, Valentina Naef, Maria Marchese, Devid Damiani, Asahi Ogi, Stefano Doccini, Baldassare Fronte, Jingyu Yan, Filippo M. Santorelli

**Affiliations:** 1 Department of Neurobiology and Molecular Medicine, IRCCS Fondazione Stella Maris, 56128 Pisa, Italy; 2 Department of Veterinary Science, University of Pisa, 56124 Pisa, Italy; 3 Laboratory of Separation Science for Analytical Chemistry, Dalian Institute of Chemical Physics, Chinese Academy of Sciences, Dalian 116023, China

**Keywords:** nutraceuticals, milk oligosaccharides, sialylated oligosaccharides, human milk, bovine milk, zebrafish, locomotor behavior, thigmotaxis, brain development, RNA-seq

## Abstract

Milk oligosaccharides are a complex class of carbohydrates that act as bioactive factors in numerous defensive and physiological functions, including brain development. Early nutrition can modulate nervous system development and can lead to epigenetic imprinting. We attempted to increase the sialylated oligosaccharide content of zebrafish yolk reserves, with the aim of evaluating any short-term effects of the treatment on mortality, locomotor behavior, and gene expression. Wild-type embryos were microinjected with saline solution or solutions containing sialylated milk oligosaccharides extracted from human and bovine milk. The results suggest that burst activity and larval survival rates were unaffected by the treatments. Locomotion parameters were found to be similar during the light phase between control and treated larvae; in the dark, however, milk oligosaccharide-treated larvae showed increased test plate exploration. Thigmotaxis results did not reveal significant differences in either the light or the dark conditions. The RNA-seq analysis indicated that both treatments exert an antioxidant effect in developing fish. Moreover, sialylated human milk oligosaccharides seemed to increase the expression of genes related to cell cycle control and chromosomal replication, while bovine-derived oligosaccharides caused an increase in the expression of genes involved in synaptogenesis and neuronal signaling. These data shed some light on this poorly explored research field, showing that both human and bovine oligosaccharides support brain proliferation and maturation.

## 1. Introduction

Milk is fundamental in the early nutrition of all baby mammals, being their primary source of nutrients and bioactive compounds [1]. Proteins, lipids, carbohydrates, minerals, and vitamins are the main milk nutrients necessary to support the newborn’s development until the weaning period [2]. Milk oligosaccharides, counted as the third component in quantity of the solid part of milk, are a complex class of glycans that have no direct nutritional value for the offspring, but act as bioactive factors in numerous defensive and physiological functions [3]. Although indigestible by infants, on reaching the large intestine, they act as prebiotics, stimulating the growth of beneficial microbiota [3,4], and as defensive agents against various toxins, pathogenic bacteria and influenza viruses, thus limiting the onset of enteric infections [5,6]. Moreover, milk oligosaccharides not only modulate the immune system [7], promoting beneficial effects on allergic disorders [3] and autoimmune diseases [8], but also contribute to brain development and cognition [9]. These molecules are synthesized in the mammary gland starting from monosaccharide units, which are combined with a lactose core through numerous possible linkages, resulting in a wide range of different structures that reflect the multitude of their biological functions [10]. Milk oligosaccharides in human breast milk have been extensively investigated and more than 200 different types have already been found [11]. On the other hand, domestic animal milk oligosaccharides have been less studied and only 48 types have been characterized in cow, goat, sheep, pig, horse, and camel milk [12]. Two classes of oligosaccharides are naturally found in milk: neutral and acidic. Chemically, the first has a fucosylated end, whereas the latter has a sialic acid terminal residue. Neutral oligosaccharides are the most common in human milk, whereas sialylated oligosaccharides account for about 80–90% of total oligosaccharides in non-primate animal milk [12]. The total sialylated oligosaccharide content of milk from traditional dairy animals is 10–100 times lower than that of human milk [12,13,14].

Sialylated milk oligosaccharides (SMOs) seem to play an essential role in brain development as they act as the main suppliers of sialic acid, an important constituent of key brain proteins as gangliosides and myelin associated glycoprotein [7,15,16] involved in the formation of brain structures sustaining cognition. In this context, it is well known that early nutrition can modulate nervous system development [17] and, through changes in gene expression patterns and nutrient-sensitive signaling pathways, can lead to epigenetic imprinting that may have lifelong effects on health status [18]. Moreover, several studies have shown that the duration of breastfeeding is the main associated variable to the infant intelligence and cognition performances [19]. Wang [20] has postulated that low levels of sialic acid may be a main disadvantage of infant formula milks. These data have been supported by brain imaging studies showing that breast milk promotes infant brain development, particularly white matter growth [21]. An increasing body of literature suggests that newborns could benefit from exogenous sialic acid supplementation in order to meet the demands of the rapidly growing brain [4], and also that several diseases of the brain (such as mental retardation, schizophrenia, and Alzheimer’s and senile dementia) are associated with lower brain sialic acid and/or ganglioside content [22,23]. It seems that intake of compounds containing sialic acid may reduce the decline in brain activity that occurs with aging [15]. Sialylation is a process essential not just for the brain, but also for normal muscle function [24] and skeletal development [25]. Importantly, while adult mammals can endogenously synthesize sialic acid from glucose and other products of glycolysis, newborns largely lack this capability, and thus require an exogenous source of sialic acid [16]. Studies in animal models suggest that administration of SMOs and/or sialic acid was associated with enhancement of specific and non-specific immunological resistance [26], increases in cerebral and cerebellar ganglioside content [13,15], and positive effects on microbiota, behavioral responses during stressor exposure [27], and cognitive abilities [20,28]. Rodents receiving neonatal exogenous supplementation of sialic acid showed improved novel object recognition memory and long-term potentiation (a cellular process thought to be associated with memory) [16,29]. Furthermore, mouse pups fed with a milk deprived of the most abundant SMOs, i.e., 6′-sialyllactose (6′SL) or 3′-sialyllactose (3′-SL), exhibited impairments in memory, attention, and hippocampal long-term potentiation [30].

In the current study, using a microinjection technique, we attempted to increase the sialylated oligosaccharide content of zebrafish yolk reserves at early stages of embryogenesis, with the aim of evaluating the short-term effects of the treatment on mortality, locomotor behavior, and gene expression. The zebrafish is already considered an ideal organism for neurobiology and genetics research and for exploring possible new drug treatments [31,32,33,34,35]. The use of zebrafish, rather than mammalian models, for genetic and drug studies offers numerous advantages, such as high fecundity, egg transparency, rapid ex utero embryogenesis (which allows noninvasive drug treatments at early embryonic stages) and affordability [36,37,38]. In this context, numerous behavioral protocols have already been validated for this species, even in its larval stage [39,40]. Despite the large differences in embryo development and nutrition between fish and mammals, sialic acid is found in all vertebrates (even birds, reptiles, and amphibians) and essentially in all tissues, with the highest concentration in the central nervous system [17]. While the delivery of external nutrients in mammal newborns is limited to maternal nutrient transfer (prenatal) and consequently to the onset of milk feeding (postnatal), in zebrafish it is possible to easily exert a nutraceutical stimulus during embryogenesis, injecting a specific compound directly into the egg yolk, prior the hatching event [18]. Research on the effects of SMOs administration on health and brain functions is still limited and no experimental data on important vertebrate models, such as zebrafish, are available in literature to the best of our knowledge.

## 2. Results

### 2.1. Locomotor and Thigmotactic Behavior

**Figure 1:**
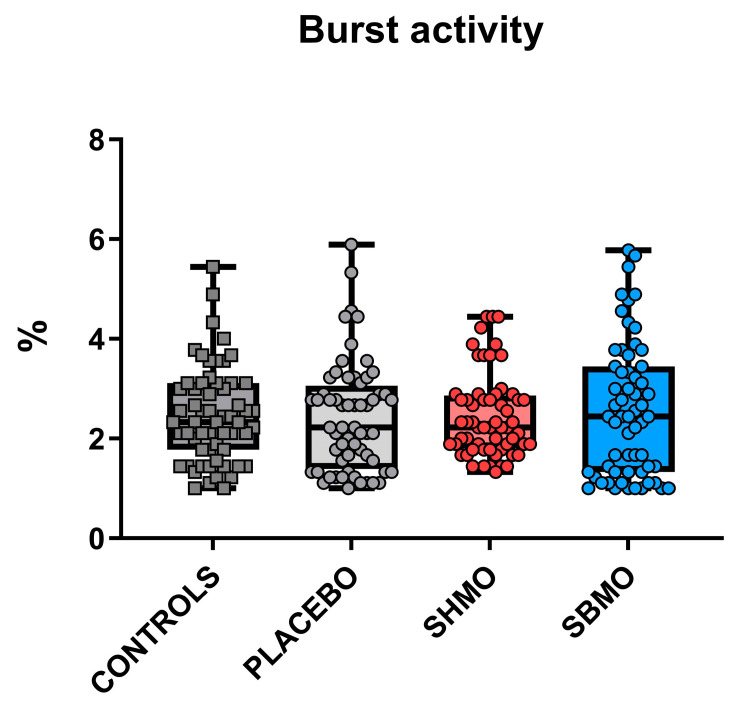
Burst activity of 30 hpf embryos. Individual values are plotted as median with 95% confidence intervals (*n* = 60 larvae per group). Controls were untreated and placebo larvae were microinjected with saline solution vehicle. Abbreviation: SHMO: sialylated human milk oligosaccharide treatment; SBMO: sialylated bovine milk oligosaccharide treatment.

As the developing nervous system presents high sensitivity to drug exposures, a developmental neurotoxicity analysis was performed on wild-type zebrafish larvae microinjected into the yolk with either sialylated milk oligosaccharides solutions (at doses equal to 100 mmol/L, purified from human or bovine milk) or saline solution (placebo group). The same analyses were performed even in untreated wild-type larvae, as the control group. For this purpose, two standardized behavioral tests were used. The first one was the tail-coiling test, which allows to evaluate the action mode of compounds interfering with neurotransmission. This test is based on the analysis of the spontaneous embryo tail coiling inside the egg, and it involves side-to-side contractions of the trunk, consisting of the first motor behavior of embryos [41]. Coiling analysis at 30 h post fertilization (hpf) showed similar burst activity between the controls and treated embryos, with no significant differences observed between groups (Figure 1).

**Figure 2:**
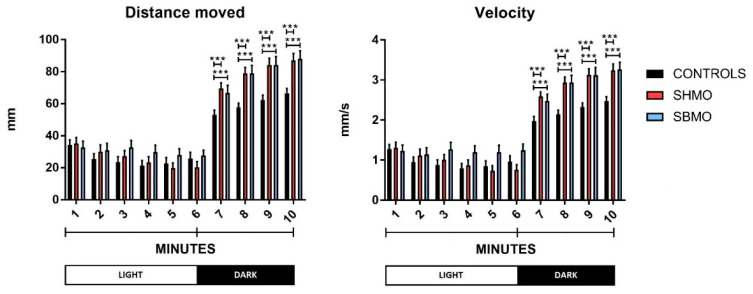
Larvae locomotion data at 5 dpf. Velocity was expressed as distance travelled (millimeters) per time unit (seconds) and measured for every single minute. Values are expressed as means (*n* = 72 larvae for each group). The error bars show the standard error of the mean. ***: *p* ≤ 0.001 calculated by Mann–Whitney U test. Abbreviation: SHMO: sialylated human milk oligosaccharides treatment; SBMO: sialylated bovine milk oligosaccharides treatment.

The second behavioral test used in this study was the visual motor response (VMR) test, carried out on hatched larvae of 5 days post fertilization (dpf). This assay allows to analyze the larvae locomotor behavior in different light conditions, with the aim of evaluating the sensorimotor function of zebrafish larvae, and to characterize neurobehavioral responses to chemicals. Typically, untreated zebrafish larvae exhibit higher locomotor activities during the dark phase than in the light phase, because a dark environment offers a better chance of survival for the larvae [42]. Indeed, calculation of average locomotor activity per minute revealed that the zebrafish larvae covered a greater distance and moved at greater speed during dark than light phases, regardless of the experimental treatment (Supplementary Figure S1). However, locomotion data from the VMR test reported in Figure 2, showed that during the dark phase, both distance and velocity of movement were found to be significantly different between the control and SMO-treated larvae. In particular, throughout the dark phase, larvae treated with both SMOs displayed increased test plate exploration, with greater distance moved and faster velocity of movement compared with the control group values.

**Figure 3:**
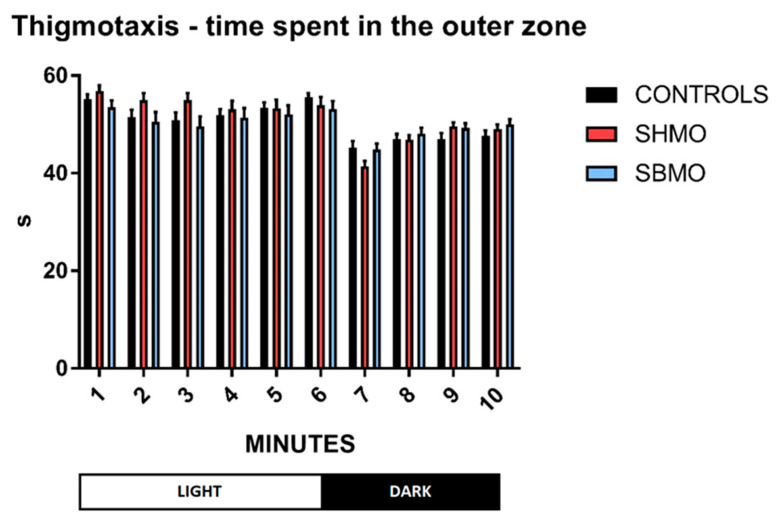
Thigmotaxis data at 5 dpf shown as time (s) spent in the outer zone of the test apparatus per minute. Values are expressed as means (*n* = 72 per each group). The error bars show the standard error of the mean. Abbreviation: SHMO: sialylated human milk oligosaccharide treatment; SBMO: sialylated bovine milk oligosaccharide treatment.

In order to highlight a potential stress response to SMO treatments, the thigmotaxis behavior, which is a well-established index of anxiety, was evaluated in both light and dark conditions, by using the data obtained from the VMR test. The thigmotaxis data showed that all larvae spent significantly more time in the outer versus the inner zone (i.e., more thigmotactic behavior) throughout the test (Figure 3), and no significant differences were found between the controls and treated groups, in either the light or dark phases.

Analysis of average thigmotactic behavior per minute showed that zebrafish larvae spent more time in the outer zone during light versus dark phases, regardless of the experimental treatment (Supplementary Figure S2). This obviously implies that all the larvae (controls and treated groups) explore the center of the well plate more during darkness.

### 2.2. Larval Survival Rate

**Figure 4:**
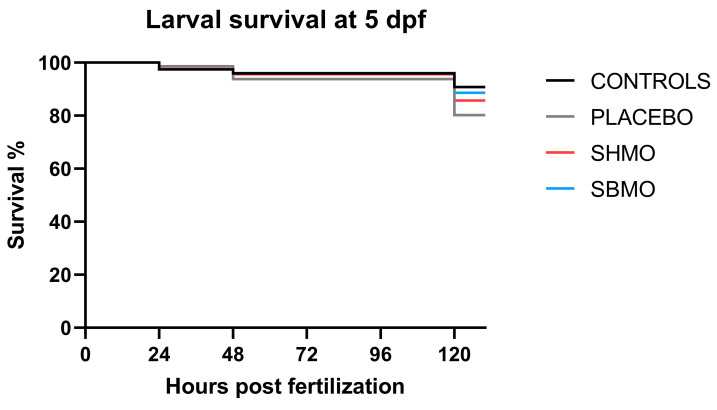
Kaplan-Meier survival curves of zebrafish larvae at 5 dpf (*n* = 300 per group). Controls were untreated and placebo larvae were microinjected with saline solution vehicle. Abbreviation: SHMO, sialylated human milk oligosaccharide treatment; SBMO, sialylated bovine milk oligosaccharide treatment.

The larval survival rate at 5 dpf was high (>80%) in all the groups of zebrafish larvae, therefore no significant differences were found between controls, placebo, and the two SMO groups (Figure 4). The highest survival rate was observed in the control group (90.59%) and the lowest in the placebo group microinjected with saline solution only (80.14%), while intermediate values were observed in the two groups treated with milk oligosaccharides: 85.96% in larvae treated with sialylated human milk oligosaccharides (SHMOs) and 88.62% in larvae treated with sialylated bovine milk oligosaccharides (SBMOs).

### 2.3. Comparative Analysis and Bioinformatic Categorization of Differentially Expressed Genes

In order to better characterize the effects of the different SMO treatments on zebrafish development, we planned to obtain transcriptomic profiles out of mRNAs taken from larvae either microinjected with human or with bovine SMOs, or from non-microinjected controls. Sequencing libraries were therefore generated from three different pools of zebrafish larvae per each experimental condition, with each pool containing 12 individuals. After sequencing, reads were trimmed, aligned, annotated on the zebrafish database, and compared for differential expression with the DESeq2 method. To allow comparison of gene expression profiles, the normalized gene abundance level of each microinjected embryo pool, compared with the profile of non-microinjected ones, was calculated, and reported as log2 fold change (log2(FC)). Transcripts showing a log2(FC) ≥ 0.58 and an adjusted *p*-value (*p*-adj) ≤ 0.05 were assigned as differentially expressed. A further profile was obtained by comparing the treatments, in order to discriminate the specificity of changes found. The transcriptomic profiles identified 1137 differentially expressed transcripts in SHMO-treated embryos (508 were up-regulated and 629 down-regulated, Supplementary Table S1), and 2296 in SBMO-treated embryos (883 up-regulated and 1413 down-regulated, Supplementary Table S2). The comparison of the two treatments showed that 879 transcripts were specifically dysregulated (248 up- and 631 down-regulated, Supplementary Table S3).

**Figure 5:**
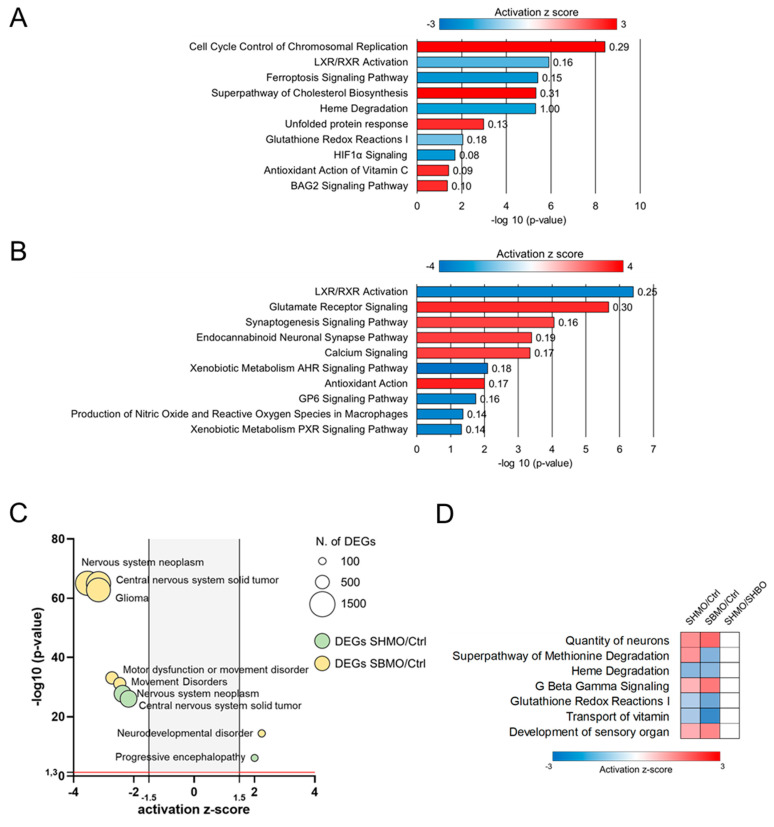
Bioinformatic analysis of RNA-seq data. Top meaningful canonical pathways in the SHMO (**A**) and SBMO (**B**) datasets were sorted by -Log(*p*-value). A positive z-score (red) denotes pathway activation, and a negative z-score (blue) denotes pathway inhibition. The numbers next to the bars are the ratios of DEGs to the total number of genes making up the pathway. (**C**) Multivariable graph showing most the representative disease annotations in both datasets. (**D**) Heat map of the comparison analysis between conditions, highlighting common disease and functional annotations between datasets.

Differentially expressed genes (DEGs) were categorized using the IPA bioinformatic suite (https://www.qiagen.com, accessed on 31 May 2022). In SHMO-treated larvae, this step revealed three different macro-categories related to: (i) embryonic development (*BAG2 Signaling Pathway*, *Cell Cycle Control of Chromosomal Replication*); (ii) redox homeostasis (*Antioxidant Action of Vitamin C*, *Glutathione Redox Reactions I*, *Unfolded Protein Response*, *Heme Degradation*, *Ferroptosis Signaling Pathway*); and (iii) regulation of lipids, glucose and cholesterol metabolism (*Heme Degradation*, *LXR/RXR Activation*, *HIF1α Signaling*, *Superpathway of Cholesterol Biosynthesis*) (Figure 5A). Instead, the SBMO transcriptomic profile showed multiple differentially expressed genes shared with the SHMO group profile, and related to: (i) nervous system development (*Glutamate Receptor Signaling*, *Endocannabinoid Neuronal Synapse Pathway*, *Synaptogenesis Signaling Pathway*, *Calcium Signaling*); (ii) redox homeostasis (*Antioxidant Action*, *Production of Nitric Oxide and Reactive Oxygen Species in Macrophages*); (iii) metabolism (*Calcium Signaling*, *LXR/RXR Activation*, *Xenobiotic Metabolism PXR Signaling Pathway*, *Xenobiotic Metabolism AHR Signaling Pathway*); and (iv) inflammatory response (*GP6 Signaling Pathway*) (Figure 5B).

Further scrutiny of the main annotations in the profiles of the SMO-treated larvae pinpointed disease and functional annotations related to nervous system, neurodevelopmental and movement disorders as the main high-ranked categories affected by the treatment (Figure 5C). For a better understanding of biological processes and diseases commonly affected by the treatments, we performed a comparison of the previously assessed core analyses, using the z-score values of common annotations. Several biological functions were severely affected in both SHMO- and SBMO-treated embryos and predicted to be activated or inhibited with a similar trend. Detailed information about expression of DEGs within the main categories can be found in Supplementary Figures S3–S21. Functions mainly related to neuronal development and free-radical scavenging were also annotated using this comparative approach (Figure 5D).

**Figure 6:**
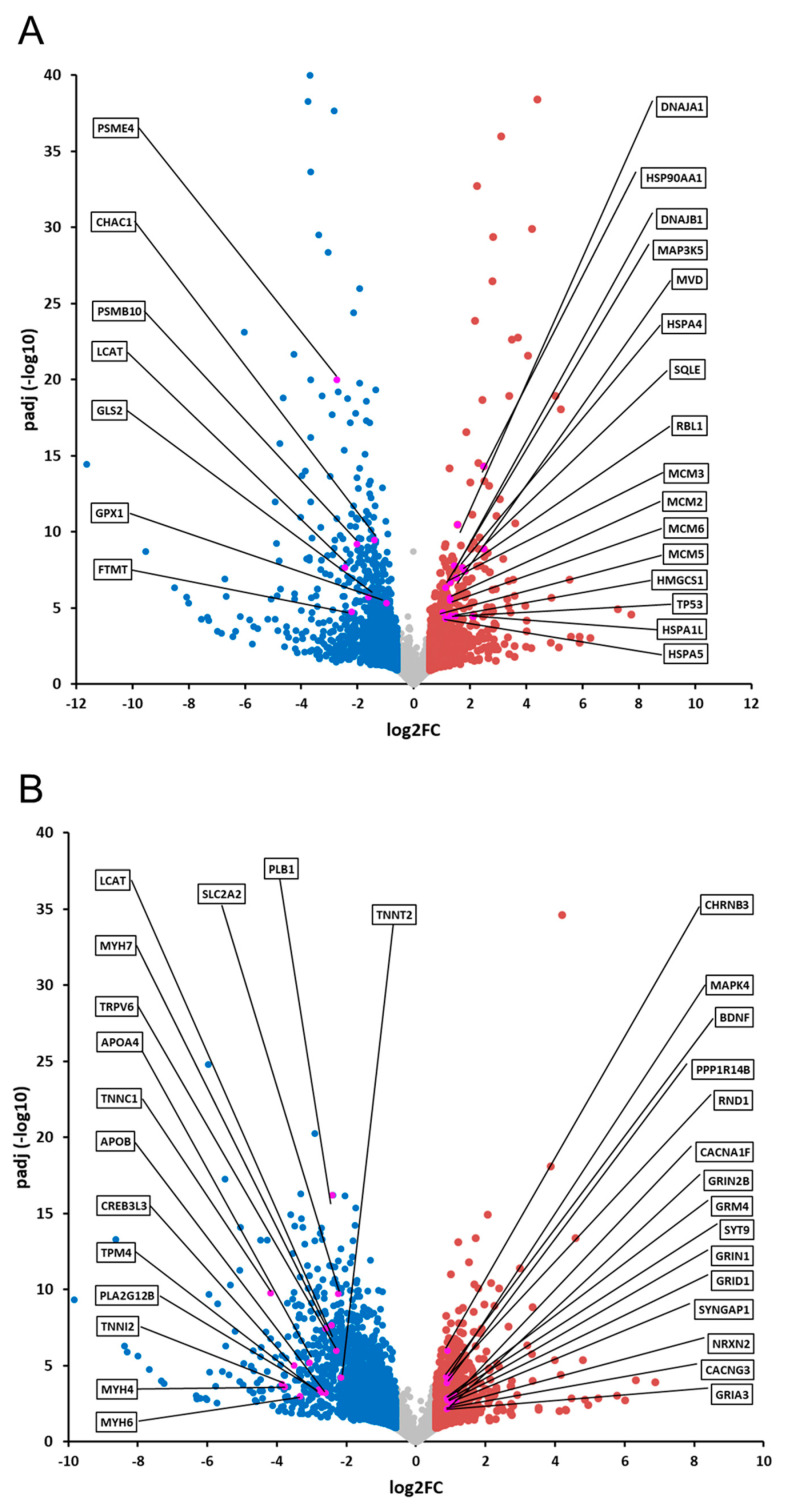
Data obtained from RNA-seq experiments for injections of SHMOs (**A**) and SBMOs (**B**) in zebrafish larvae are represented as Volcano plots. DEGs are colored in blue if down-regulated and red if up-regulated. In evidence (purple dots) are depicted for selected genes belonging to top meaningful pathways, as retrieved from IPA analysis (Figure 5A,B).

Whole datasets from RNA-seq experiments related to the two different treatments on zebrafish larvae are also represented as Volcano plots (Figure 6).

**Figure 7:**
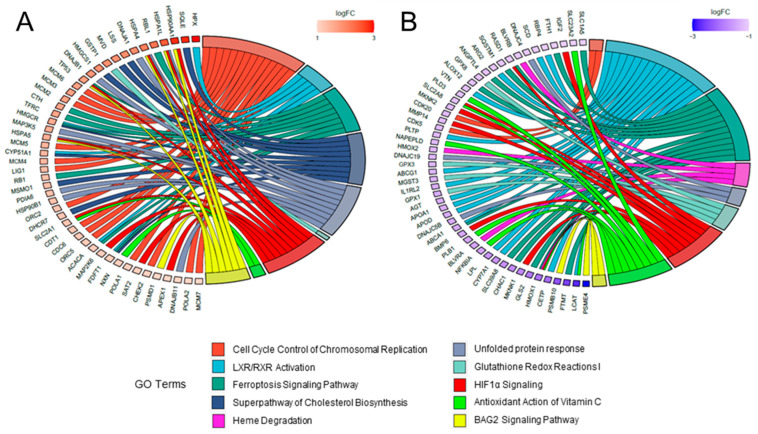
Schematic representation of DEGs in the SHMO injection in zebrafish larvae. Top50 upregulated (**A**) and down-regulated (**B**) genes in top meaningful canonical pathways (as depicted in Figure 5A), represented as GO chord plot. The intensity of the box adjacent to the gene name indicates the level of expression, as shown in the log(FC) legend.

**Figure 8:**
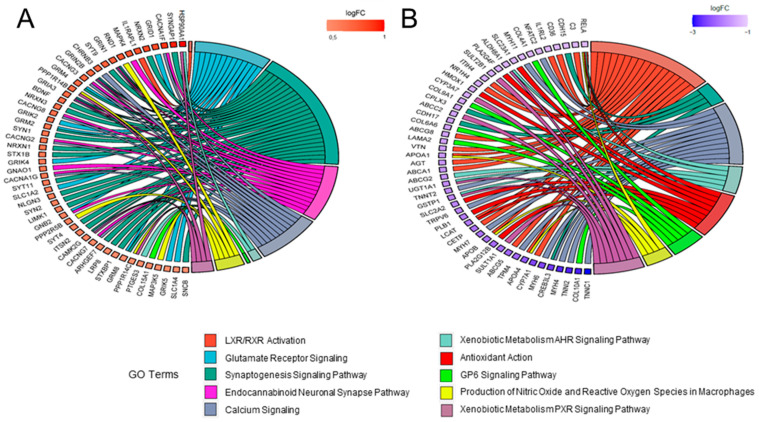
Schematic representation of DEGs in SBMO injection in zebrafish larvae. Top50 up-reg-ulated (**A**) and down-regulated (**B**) genes in top meaningful canonical pathways (as depicted in Figure 5B), represented as GO chord plot. The intensity of the box adjacent to the gene name indicates the level of expression, as shown in the log(FC) legend.

In addition, the relationship between DEGs and main categories were reported with gene ontology (GO) chord plots, also allowing graphical representation of the level of expression of single genes. In particular, the top50 up- and down-regulated genes for both the SHMO (Figure 7A and Figure 7B, respectively) and SBMO treatment (Figure 8A and Figure 8B, respectively) were taken into account for the analysis. The differential effect of the two treatments was clearly shown in these plots. For instance, DEGs belonging to the class of *Cell Cycle Control of Chromosomal Replication* were mostly up-regulated in the SHMO treatment, together with genes related to *BAG2 Signaling* (Figure 7A). In the SBMO treatment, instead, up-regulation occurs mostly for *Glutamate Receptor* and *Calcium Signaling* pathways (Figure 8A), with not even one DEG in the first category reported as downregulated (cyan chords are absent in Figure 8B; for details, also see Supplementary Figures S3–S21).

## 3. Discussion

Sialylated milk oligosaccharides seem to play an essential role in brain development, and it is well known that early nutrition can modulate nervous system development [17] and can have lifelong effects on health status. It has been hypothesized that adequate intake of sialic acid is crucial for infant brain growth and development [43]. In this work, we used zebrafish embryos as a model to study the effects of milk-derived sialylated oligosaccharides on embryo development. The SHMOs and SBMOs were injected into fertilized zebrafish eggs, and larvae were collected at 5 dpf for behavioral and transcriptomic analyses.

Zebrafish embryos develop rapidly inside the eggs and show their first spontaneous movements at an early stage of development. This locomotor behavior begins at 17 hpf, peaks at 19 hpf, and then decreases gradually thereafter [44]. It has recently been suggested that the period from 26 to about 30 hpf is characterized by relative stability of all embryo coiling activity parameters, analysis of which could constitute a rapid new way to screen for developmental neurotoxicity induced by drugs [45]. Analysis of tail flicks at 30 hpf in treated embryos did not show significant alterations in burst activity (i.e., the percentage of time an embryo is moving) compared with controls, excluding a putative neurotoxicity effect of used milk oligosaccharides. Furthermore, the current study seems to confirm the appropriateness for zebrafish embryos of the chosen injection timepoint (4.7 hpf) and volume (4.6 nL), given that no negative effects on survival or development were recorded. In fact, survival rates were similar across controls, placebo and both SMO treatment groups, and comparable with the results obtained by other researchers [18,46].

Locomotor behavior analysis was performed using the VMR test, which is based on “a stereotypical series of larval motor responses provoked by changes in ambient illumination” [47]. The VMRs triggered by drastic changes in illumination could be elicited both at light onset and at light offset [48]. The VMR at light offset has been shown to consist of a significant increase in locomotion for about 30 min, after which locomotor activity returns to the baseline level [49]. Conversely, at light onset, the increase in locomotion lasts for about 30 s [49]. Hyperlocomotion upon sudden transition to the light condition has been attributed to increased stress/anxiety [50]. That said, regardless of the stimulus-response model, zebrafish larvae typically exhibit higher locomotor activity during dark phases [51,52], as occurs in mice [30]. Accordingly, all our three experimental groups of 5 dpf larvae showed a significant increase in locomotor activity during dark compared with light exposure. Moreover, no group showed a decrease in locomotor activity on the light-dark transition (a response that could indicate possible sedation or tissue damage) [53]. During the dark phase, both SHMO- and SBMO-treated larvae showed enhanced locomotor behavior (distance moved and velocity) compared with the control group. Therefore, in accordance with Basnet and colleagues [50], we could possibly argue that the light-dark transition seemed to induce a clearer stress response in treated compared with control group larvae. Nevertheless, this argument is contradicted by the results of our analysis of thigmotaxis, which is a well-established sign of anxiety-like behavior [54]: we did not observe any increase for time spent in the outer zone by treated larvae in either phase. A possible explanation for these contrasting data is that the drastic change in illumination produced not an enhanced escape behavior in response to a threatening stimulus, but rather an enhancement of exploratory behavior, with the larvae seeking a dark environment that offers a better chance of survival [54]. Furthermore, even allowing for the stress response, we could argue that our results could be ascribed to an improvement in coping behavior, i.e., the automatic action taken to deal with a threatening stimulus. In line with our results, the intake of 6′SL during the early life of mice was associated with changes of locomotor activity [30]. Unfortunately, a discrimination between light and dark phase was not carried out in this latest study on rodents. However, adopting a comprehensive approach, these authors observed that behavioral alterations are associated with modifications of the serotoninergic system.

According to our RNA-seq data, both treatments seemed to exert antioxidant effects (e.g., vitamin C action) in developing fish. Nevertheless, striking differences were observed between the two treatments. The SHMOs seemed to increase expression of genes related to cell cycle control and chromosomal replication. The analysis also showed increased expression of genes related to both *Unfolded Protein Response* (UPR) and *BAG2 Signaling Pathway*, and an anti-apoptotic action. This suggests that SHMO supplementation could play an important role in supporting a high proliferation rate, as in the developing cerebral cortex for example. Rapid increases in cell numbers in tissues of this kind need to be accompanied by strict regulation of the folding of newly produced proteins, to ensure their proper functioning, together with close control of the genomic identity of daughter cells, so as to avoid increases in cells with imbalanced numbers of chromosomes [55]. Thus, decreased expression of UPR-related genes has indeed been found to induce premature neurogenesis, leading to microcephaly [56], and a reduction in the cortical size is typically seen in several models with alteration of replicative forks and/or mitotic checkpoints [57,58,59,60]. On the other hand, the zebrafish larvae treated with SBMOs seemed to display a quite different transcriptomic profile, with increased expression of genes involved in synaptogenesis and neuronal function such as glutamate receptors and genes involved in calcium signaling and endocannabinoid signaling pathways. Interestingly, the discrepancies observed might reflect species-specific features of human and bovine newborn brains. In the former, the brain tissue proliferation rate remains high, as shown by the dramatic growth occurring during the peri- and postnatal periods, especially in the cerebral cortex [61,62,63]. For this reason, the human newborn can be considered an external fetus. Conversely, calves might require higher energy because they need to have the ability to stand and run within minutes of birth, a function requiring full maturation of neural circuits. Differences on RNA-seq data between the SHMO and SBMO groups may also be due to the different concentration of 3′SL and 6′SL in human and bovine milk.

According to our results, investigating the long-term consequences of a 6′SL-deficient milk in mice, it was found that maternal 6′SL adjusts cognitive development through a short-term up-regulation of genes modulating formation and patterning of neuronal circuits in the prelimbic medial prefrontal cortex (PFC) [30]. Interestingly, gene expression differences were not observed in the hippocampus (neither at eye-opening nor in adulthood) and the PFC-specific differences nearly vanished in adulthood. Recently, a connection between gene expression and behavior was reported in zebrafish larvae exposed to drugs of abuse [64]. According to these latter authors, the exposure to drugs may affect behavioral outcomes, but it was difficult to predict the direction of the effect. In particular, the developmental exposition to drug caused a dysregulation of the locomotor behavior and a differential expression of the innate immune genes, immediate early genes (regulators of synaptic plasticity) and circadian genes, essentially due to neuroinflammation. In our opinion, both SHMO- and SBMO-treated larvae, showing an enhanced locomotor behavior during the dark phase and an increased expression of genes involved in cell cycle control, chromosomal replication, synaptogenesis, and neuronal function, could be considered more adventurous and/or less scared compared with the control group individuals. However, the impact of SMO administration needs further mechanistic investigation.

## 4. Materials and Methods

### 4.1. Zebrafish Care and Maintenance

Adult wild-type (WT) AB strain fish were maintained at the Neurobiology and Molecular Medicine facility at the IRCCS Stella Maris Foundation (Pisa, Italy) according to standard procedures [65]. Zebrafish eggs were obtained from the natural spawning of 8-month-old adults. Once collected, fertilized eggs were incubated at 28° C in petri dishes (Ø 10 cm) filled with 50 mL of egg water (60 mg of “Instant Ocean^®^” sea salt added to 1 L of distilled water), with an initial stock density of 100 eggs per dish. Sea salt used for the preparation of the egg water was purchased from Spectrum Brands (Blacksburg, VA, USA). Handling of zebrafish complied with the guidelines of our institution’s internal animal care committee, and experiments were performed under the supervision of the Animal Care and Use Committee of the University of Pisa, in accordance with European Directive No. 63 of 22/09/2010 on the protection of animals used for scientific purposes. Every effort was made to minimize both animal suffering and the number of animals needed to collect reliable scientific data.

### 4.2. Milk Oligosaccharide Purification and Analysis

Human and bovine milk samples, each with a volume of about 20 L, were employed by the laboratory of the Dalian Institute of Chemical Physics (Chinese Academy of Sciences, Dalian, China) to obtain the milk oligosaccharide fractions used in the current study. The whole isolation-separation process was carried out in accordance with Li et al. [66]. Briefly, both milk samples, initially stored at −40 °C, were first thawed at 4 °C and then filtered using a 750 KDa and a 50 KDa hollow fiber membranes (Shandong Bona Biology, Shandong, China) to remove most of the lipids and proteins. Subsequently, the filtrates, mainly containing lactose and oligosaccharides, were injected into an electrostatic repulsion hydrophilic interaction chromatography preparation column, in order to separate the SMO fraction from lactose and neutral oligosaccharides, following Yan et al. [5]. An ACQUITY Ultraperformance system, coupled with a Xevo TQ-XS Triple Quadrupole Mass Spectrometry (Waters Corporation, Milford, MA, USA), was used to analyze the oligosaccharide samples obtained. The chromatographic separation was performed on an ACQUITY BEH Amide column with a pore size of 130 Å (particle size: 1.7 μm; inner diameter: 2.1 mm; length: 150 mm) (Waters Corporation). Reagents and standards were obtained from Sigma–Aldrich (Saint Louis, MI, USA) and pure water (18.2 MΩ) was generated by an ELGA ultrapure water system from Veolia Water Technologies (Birmingham, UK). The composition of the two mobile phases were: 5 mmol/L ammonium acetate in acetonitrile, and 5 mmol/L ammonium acetate in pure water. The elution program was performed as follows: 7 min linear gradient from 30% (*v*/*v*) to 80% (*v*/*v*); 2 min isocratic 80% (*v*/*v*) for column washing; back to 30% (*v*/*v*) and retain 1 min for column equilibrium. The main oligosaccharides in both milks were 3′-SL [Neu5Acα(2,3)Galβ(1,4)Glc] and 6′-SL [Neu5Acα(2,6)Galβ(1,4)Glc]. The specific quantification of 3′SL and 6′SL for the SHMO sample was equal to 141.10 and 210.57 mg/g respectively, while the SBMO contained mainly 3′SL (207,30 mg/g of 3′SL and 25.88 of 6′SL).

### 4.3. Microinjection of SMOs

For the microinjection procedure, embryos were handled as described by Schubert et al. [67]. Briefly, zebrafish embryos were aligned at the edge of a microscope slide, placed in a petri dish, so that the eggs were immobilized during the microinjection procedure. Microinjection into the yolk was performed under a Leica M205FA stereo-microscope (Leica, Wetzlar, Germany) at 4.7 hpf, according to Rocha et al. [18], with 4.6 nL of either saline solution (Danieau solution: 58 mmol/L NaCl, 0.7 mmol/L KCl, 0.4 mmol/L MgSO_4_, 0.6 mmol/L Ca(NO_3_), 2.5 mmol/L Hepes, pH 7.6) or SMO solutions. The SHMO and SBMO solutions were each prepared with the corresponding purified SMO fraction dissolved in the Danieau solution, to obtain a concentration of 100 mmol/L. The chosen concentration was based on the maximum solubility of the SMOs in the Danieau solution. Typically, the microinjection procedure in zebrafish is used for the inclusion of RNA/DNA, antisense morpholinos or CRISPR/Cas9, and the period selected for performing microinjection range from 1- to 2-cell stages, according to standard procedures [65]. In this study, the embryo microinjection was performed at 4.7 hpf (approximately at 30% epiboly stage) since it has been demonstrated that injection into the yolk during the epiboly stage leads to successful diffusion of the injected material within the yolk without later outward flux [18]. Furthermore, at the 30% epiboly stage the yolk syncytial layer (implicated in transporting nutrients from the yolk to the embryonic cells and later to larval tissues) is already formed [18].

### 4.4. Locomotion Analysis

**Figure 9:**
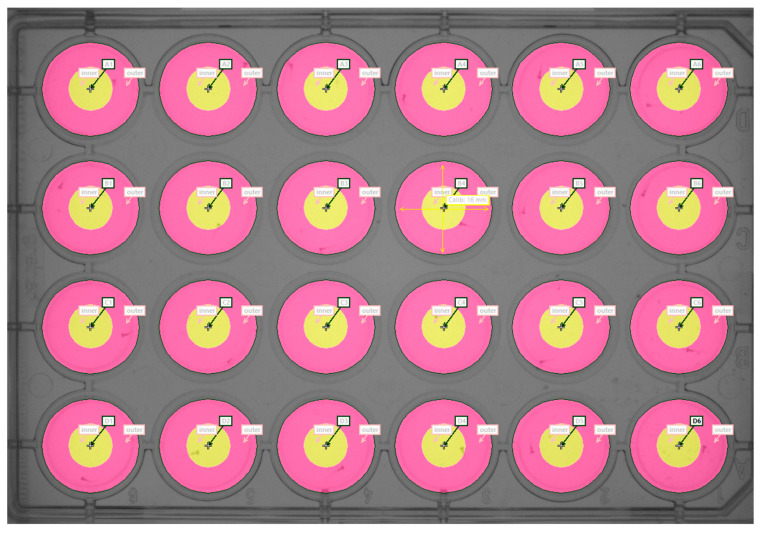
Locomotion and thigmotaxis test plate. The testing device consists of each well of the 24-well plate (diameter 16 mm for each well). The inner (yellow) and outer (pink) zones were delineated before the beginning of the experiments, using Ethovision XT12 software to draw the contours of the two zones.

In embryos at 30 hpf, coiling behavior was measured as recently described by Iacomino et al. [68] using the Danioscope software (Noldus Information Technology, Wageningen, The Netherlands). At 120 hpf, larval locomotion (distance and velocity) and thigmotactic behavior were measured using the Daniovision system connected with Ethovision XT12 (Noldus Information Technology), a specific video tracking software. Briefly, single larvae were taken from the rearing dishes and transferred into a 24-well plate along with 1 mL of egg water per well (1 larva/well). The plate was then placed in the DanioVision system and larval behavior was monitored for a total of 10 min, following Schnörr et al. [54]. The procedure was performed in two steps, including a 6-min acclimatization phase (minutes 0–6) and a 4-min interaction phase (minutes 7–10). In the acclimatization period, lights were kept ON (intensity level: 100%) and at minute 7 lights were turned OFF abruptly and were kept OFF until the end of the procedure. In order to measure thigmotaxis, a distinction was made between the inner and outer zone of each well. The width of the outer zone was set at 4 mm from the border of the well, while the diameter of the inner zone was set at 8 mm (see Figure 9).

### 4.5. Behavioral Endpoints

All swimming patterns were recorded automatically, and the following behavioral endpoints were measured:

1. Burst activity during 30 s of the test procedure, expressed as %.
2. General locomotor activity: this was measured as the total distance moved (mm) and velocity (mm/s) of movement over the whole area of the well during each minute of the test procedure, and under different conditions (lights ON vs. lights OFF).
3. Thigmotaxis: this was presented as time spent (s) in the outer zone of the well during each minute of the test procedure, and under different conditions (lights ON vs. lights OFF).

### 4.6. RNA-Seq Analysis

The whole transcriptomic analysis was carried out on zebrafish embryos microinjected with SMO solutions. Specifically, we investigated both SHMO- and SBMO-microinjected embryos. The WT non-microinjected embryos were also analyzed as a reference (Ctrl). Total RNA was extracted from larvae at 5 dpf (36 larvae per group) using the Quick RNA miniprep kit (ZymoResearch, Irvine, CA, USA), according to the manufacturer’s instructions, and checked for purity using NanoPhotometer^TM^ Pearl, version 1.2 (IMPLEN, Westlake Village, CA, USA); integrity (RNA integrity number > 7) was assessed using the RNA 6000 Pico Kit on a Bioanalyzer 2100 (Agilent Technologies, Santa Clara, CA, USA). Indexed cDNA libraries were prepared from 350 ng of total RNA using the TruSeq Stranded kit (Illumina, San Diego, CA, USA), quantified by real-time PCR, pooled at equimolar concentration, and sequenced with Illumina technology applying standard manufacturer protocols. The quality of reads was assessed using FastQC software Version 0.11.9 (http://www.bioinformatics.babraham.ac.uk/projects/fastqc/, accessed on 5 May 2022). Raw reads with more than 10% of undetermined bases or more than 50 bases with a quality score < 7 were discarded. Subsequently, reads were clipped from adapter sequences using Scythe software Version 0.994 (https://github.com/vsbuffalo/scythe, accessed on 5 May 2022), and low-quality ends (Q score < 20 on a 15-nt window) were trimmed with Sickle (https://github.com/vsbuffalo/sickle, accessed on 5 May 2022). Table S4 shows the number of sequenced and trimmed fragments. Filtered reads were aligned to the current zebrafish reference genome assembly (GRCz11) using the STAR aligner (http://code.google.com/p/rna-star, accessed on 5 May 2022).

### 4.7. Bioinformatic Analysis and Categorization of Transcriptomic Data

Within the comparisons, i.e., the SHMO treatment vs. Ctrl, SBMO treatment vs. Ctrl, and SHMO treatment vs. SHMB treatment, sets of DEGs (corresponding to identified transcripts) were evaluated by QIAGEN’s Ingenuity^®^ Pathway Analysis (IPA^®^, Spring Release, April 2022), to identify biological processes and disease and functional annotations related to a specific treatment. Specifically, we performed three independent core analyses, based on gene FC to calculate directionality (z-score) of dysregulated pathways, whereas the Ingenuity Knowledge Base reference set was used for *p*-value calculation. The most meaningful functional annotations (*p*-value < 0.05; z-score ≥ 1.5) were taken into account to estimate the predicted pathway activation or inhibition, and graphically represented. Moreover, to investigate whether significant DEGs found in the different experimental conditions might have a potential modifier role in both locomotion and neurodevelopment, we filtered disease and functional annotations, selecting only those involved in movement disorders, neurological diseases, and neurodevelopment, and reported with a z-score ≥ 1.5 for SMO vs. Ctrl analyses (but not for SHMO vs. SBMO ones). For both SHMO and SBMO vs. Ctrl analyses, GO chord plots were plotted by using a free online platform for data analysis and visualization: https://www.bioinformatics.com.cn/en, accessed on 19 July 2022.

### 4.8. Statistical Analysis

All data were analyzed applying either parametric or non-parametric methods, depending on the distribution of the response variable in question, shown by the Shapiro–Wilks test. Homogeneity of variance was assessed using the Levene test. Post-hoc comparisons were performed using the Mann–Whitney U test with Bonferroni’s correction, or an unpaired *t*-test following non-parametric analysis of variance. All statistical analyses were performed using GraphPad Prism (GraphPad Software, Inc., CA, USA).

## 5. Conclusions

In this work, we used zebrafish as a model to study the effects of milk-derived sialylated oligosaccharides on embryo development. The study confirms that the administration of SMOs extracted from human and bovine milk seems to be safe, given that no negative effects on survival or development were observed. Larval locomotor behavior analysis suggest that SMOs supplementation produced an enhancement of exploratory behavior in the dark environment, which offers a better chance of survival for the little fishes. The RNA-seq data showed that SMO-treatments pinpointed disease and functional annotations related to nervous system, neurodevelopmental and movement disorders as the main high-ranked categories affected by the treatment. In particular, SHMO supplementation could play an important role in supporting a high cell proliferation rate, as occurs in the developing cerebral cortex. On the other hand, SBMO treatment seemed to display a quite different transcriptomic profile, with increased expression of genes involved in synaptogenesis and neuronal function, such as glutamate receptors and genes involved in calcium signaling and endo-cannabinoid signaling pathways. Interestingly, the discrepancies observed might reflect species-specific features of human and bovine newborn brains. However, the impact of SMO administration needs to be explored for longer periods, from embryo to adulthood. In addition, new data on zebrafish brain morphology and imaging could be of interest to better evaluate SMO supplementation on neurodevelopment, also in disease conditions.

## Data Availability

The data presented in this study are available on request from the corresponding author. The RNA-seq data discussed in this publication have been deposited in NCBI’s Gene Expression Omnibus and are accessible through the GEO Series accession number GSE218857 (https://www.ncbi.nlm.nih.gov/geo/query/acc.cgi?acc=GSEGSE218857).

## Supplementary Materials

The following supporting information can be downloaded at: https://www.mdpi.com/article/10.3390/ijms24065456/s1.
