# Supplementary material for: Short-Term Effects of Human versus Bovine Sialylated Milk Oligosaccharide Microinjection on Zebrafish Larvae Survival, Locomotor Behavior and Gene Expression
Source: Int J Mol Sci. 2023 Mar 13;24(6):5456. doi: 10.3390/ijms24065456 (PMC10051688; doi:10.3390/ijms24065456)

## Slide 1
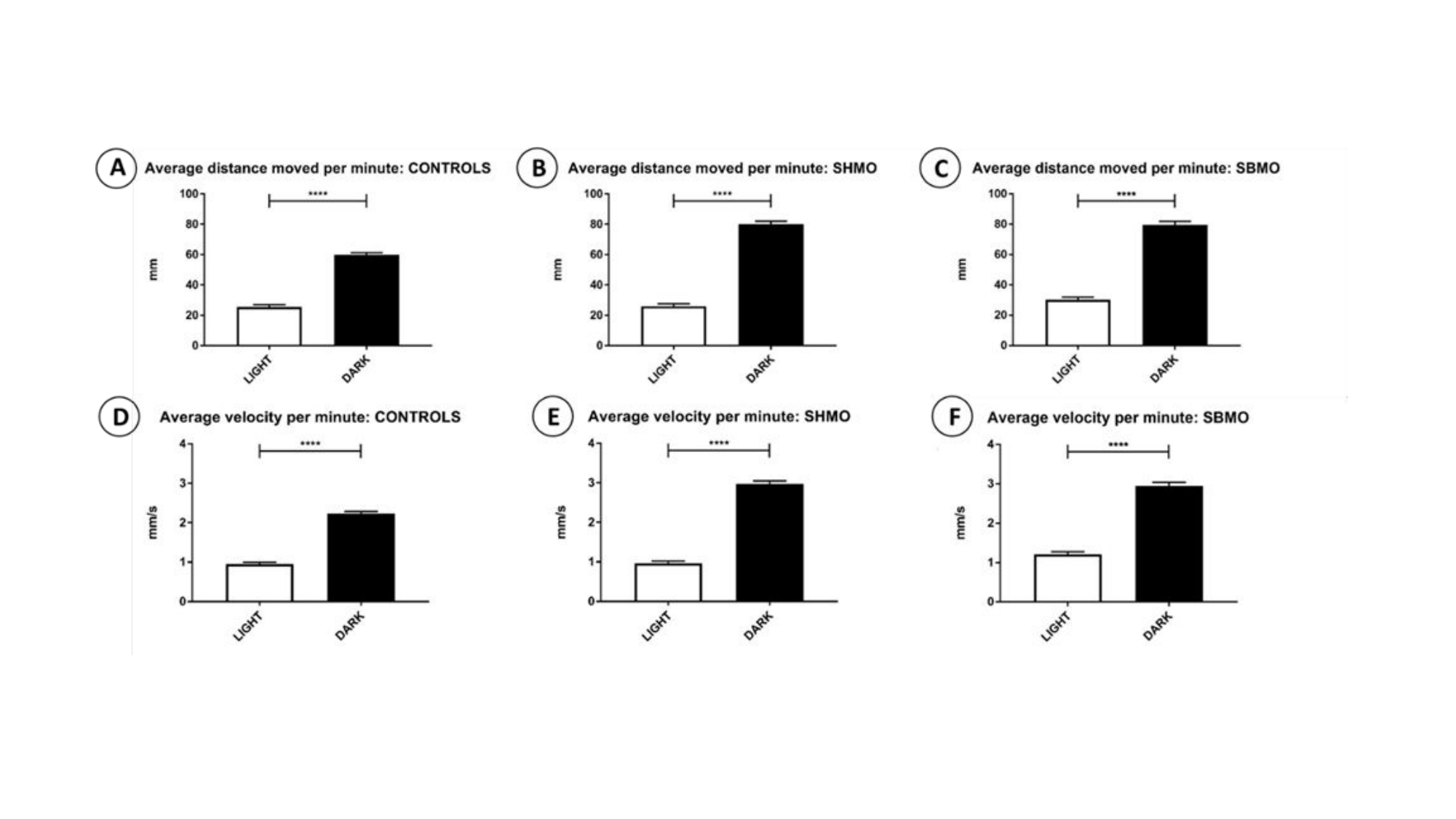

## Slide 2
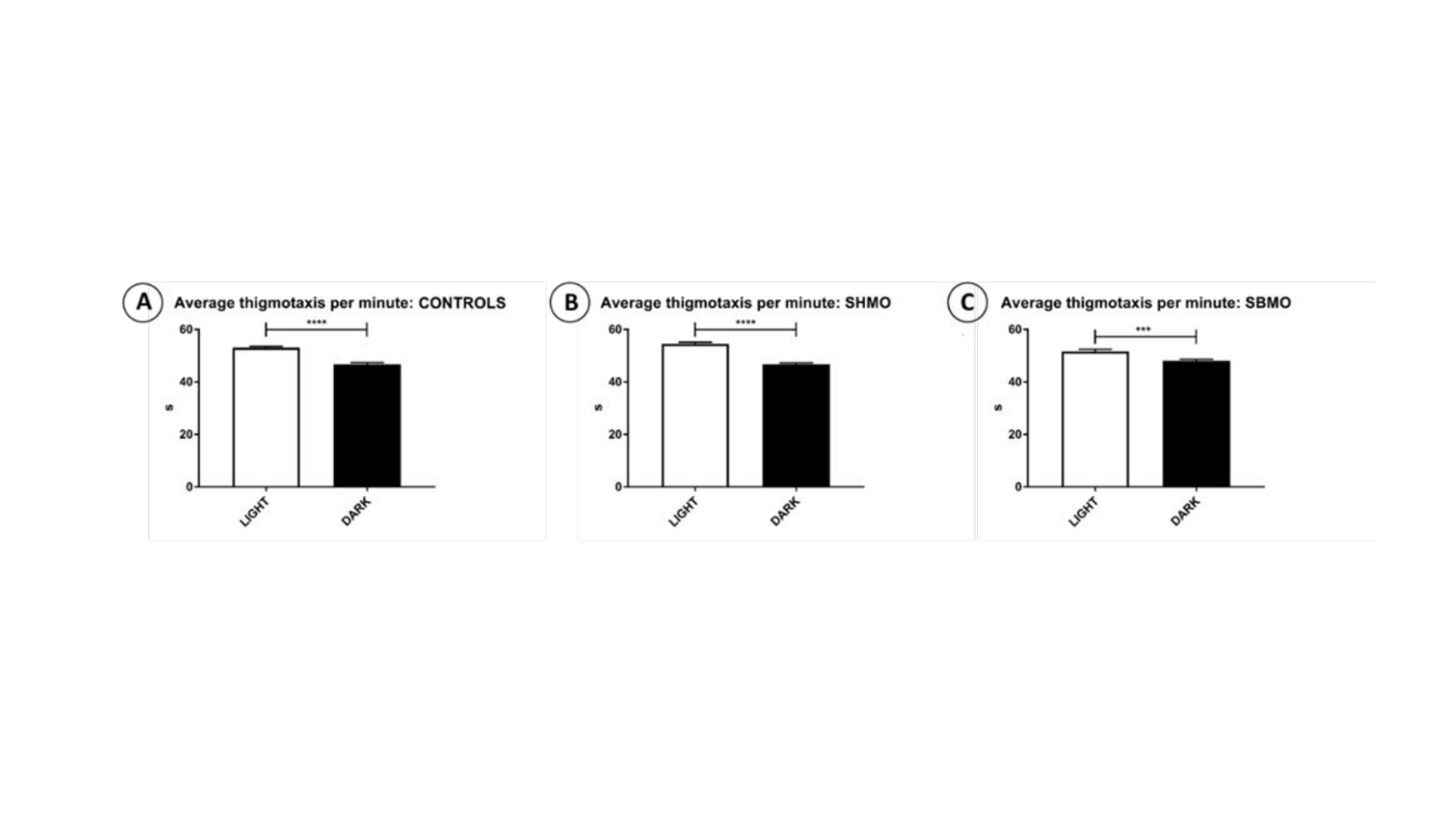

## Slide 3
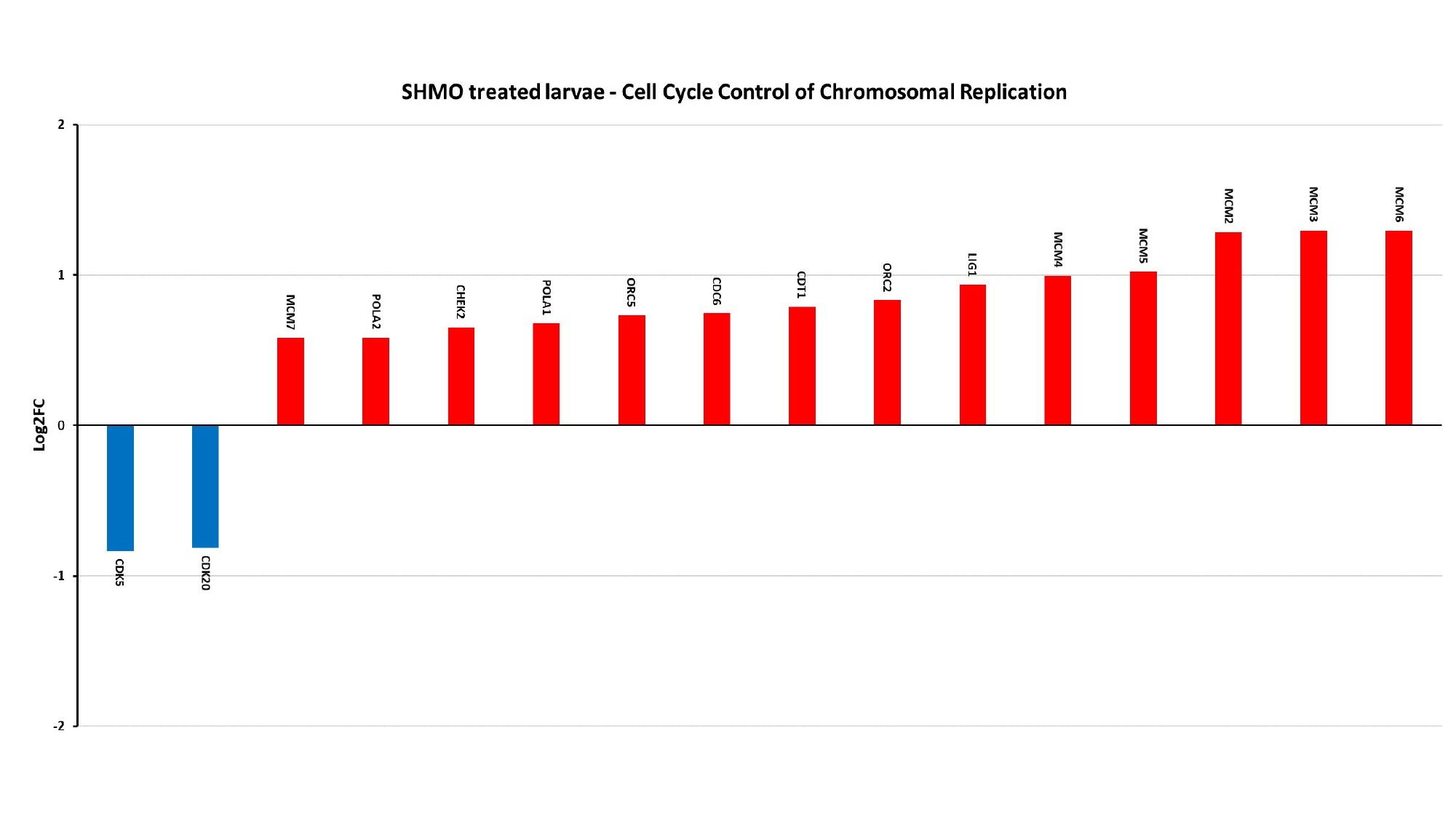

## Slide 4
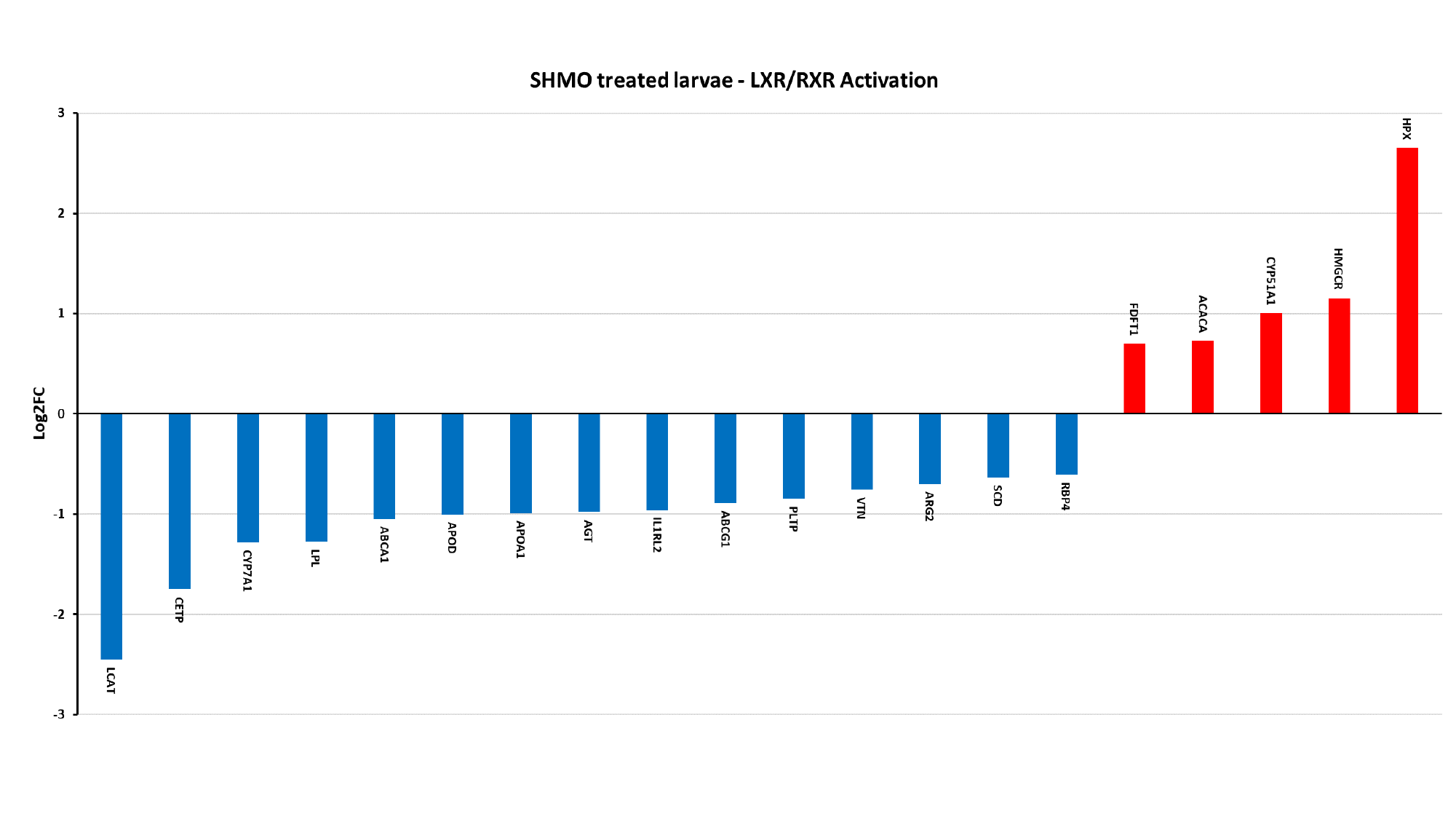

## Slide 5
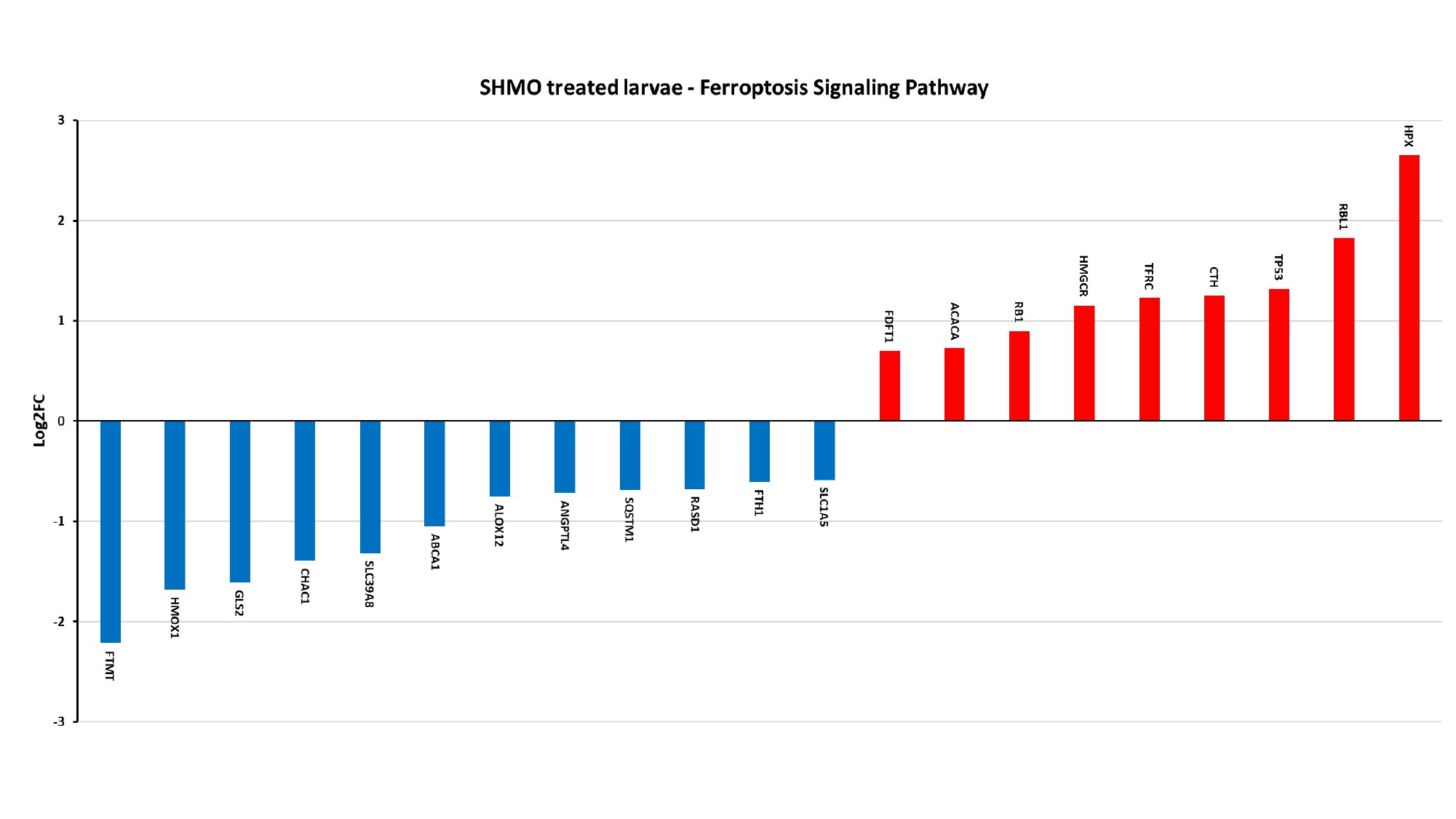

## Slide 6
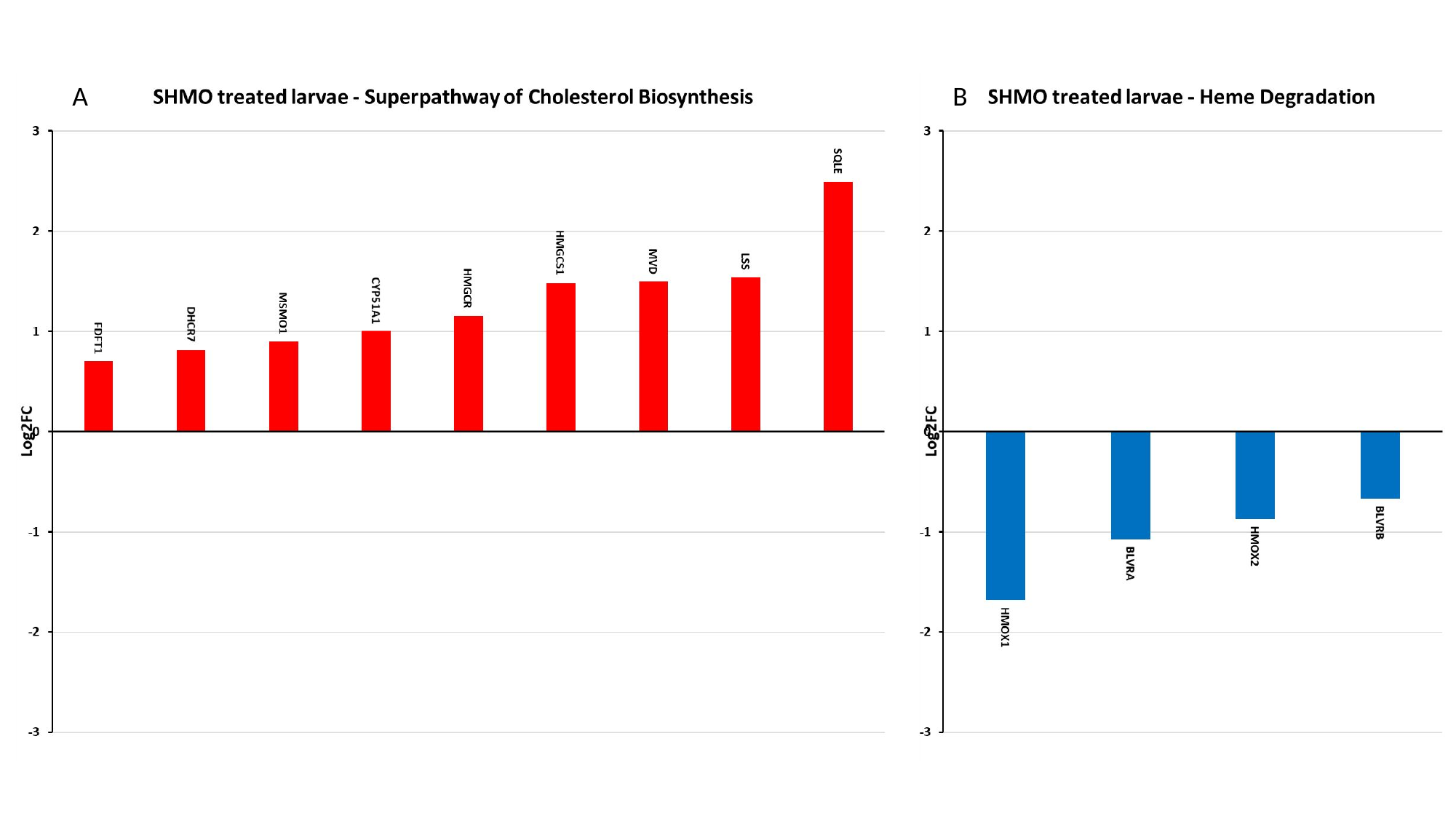

A
B

## Slide 7
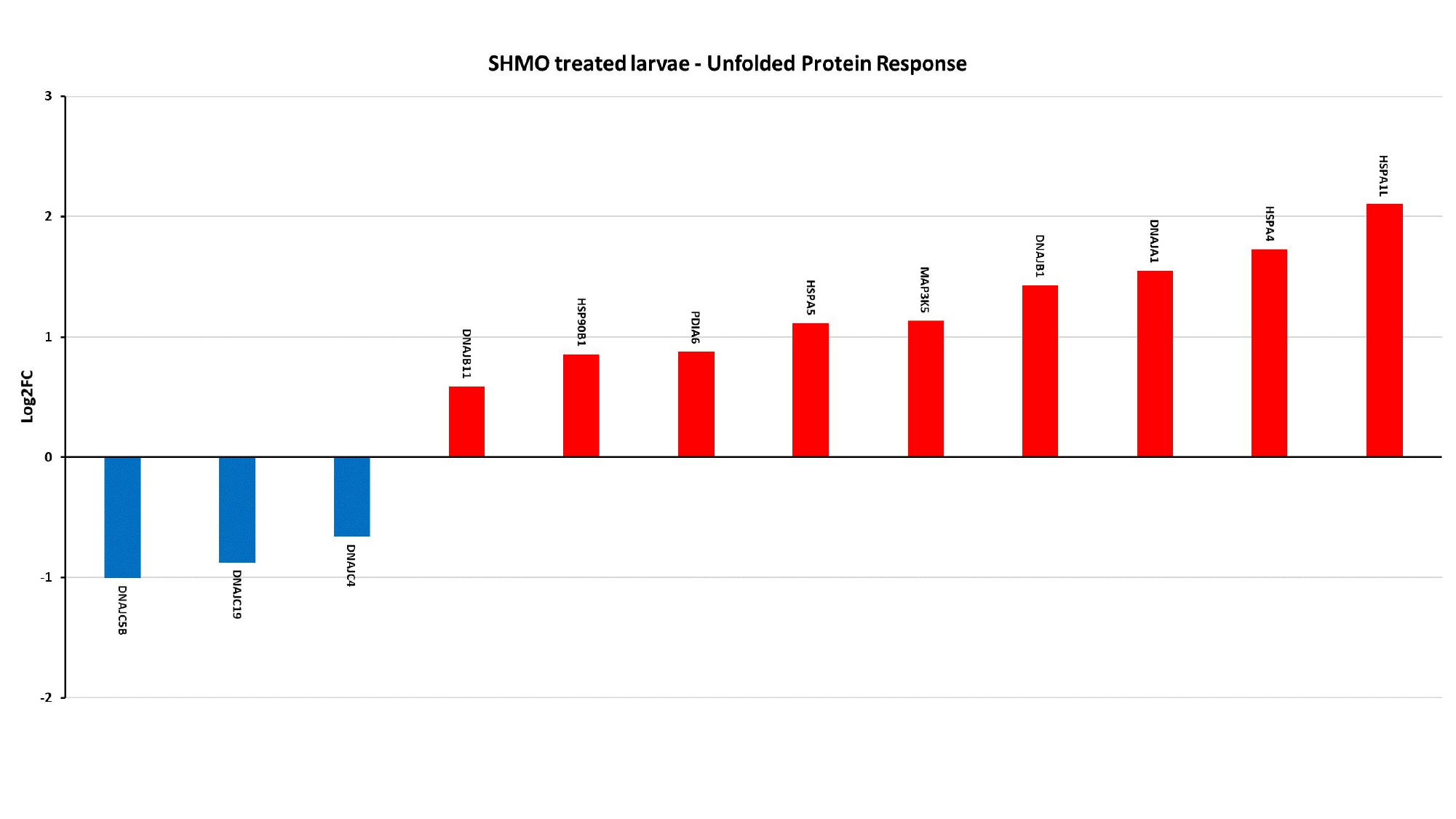

## Slide 8
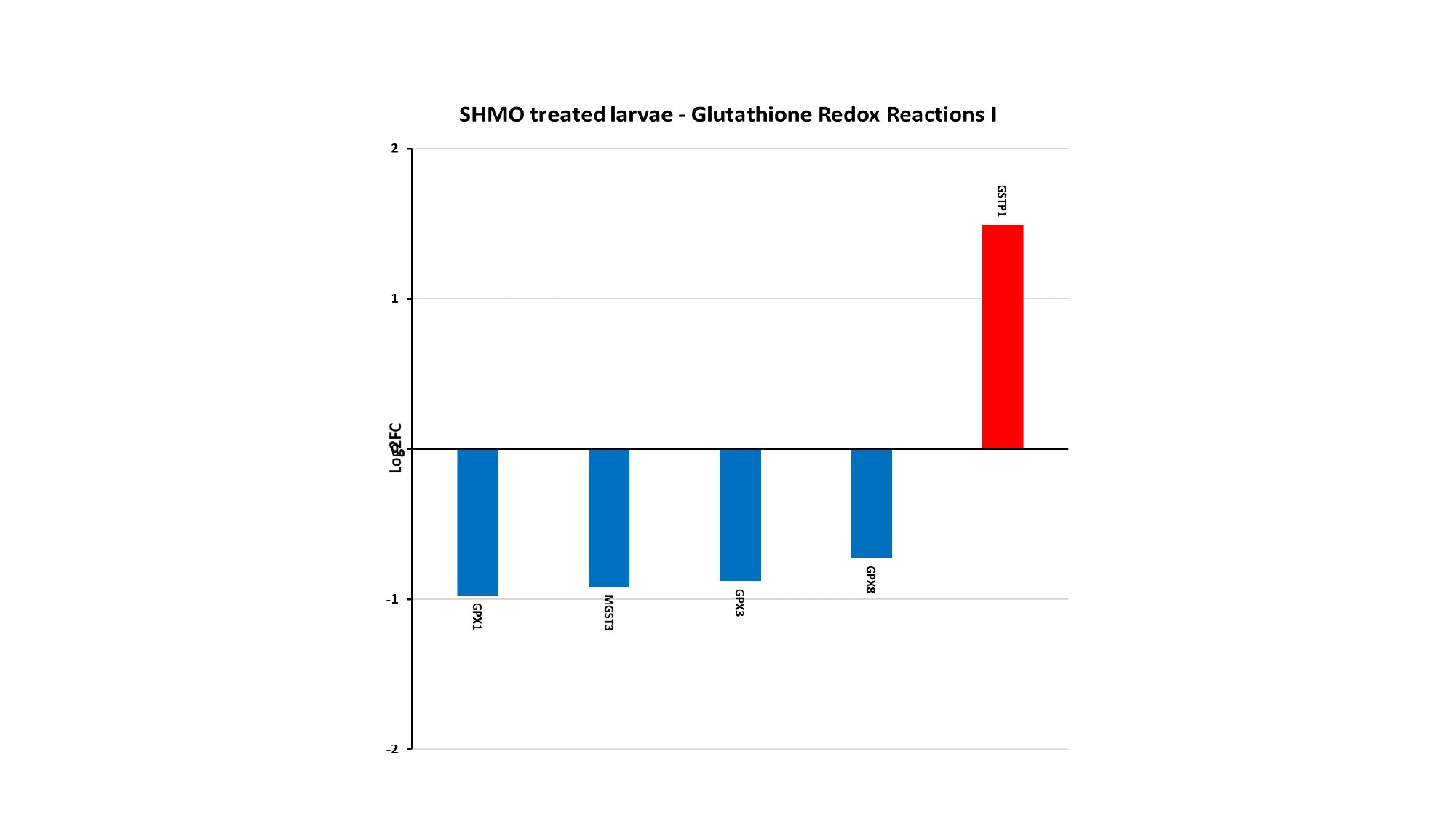

## Slide 9
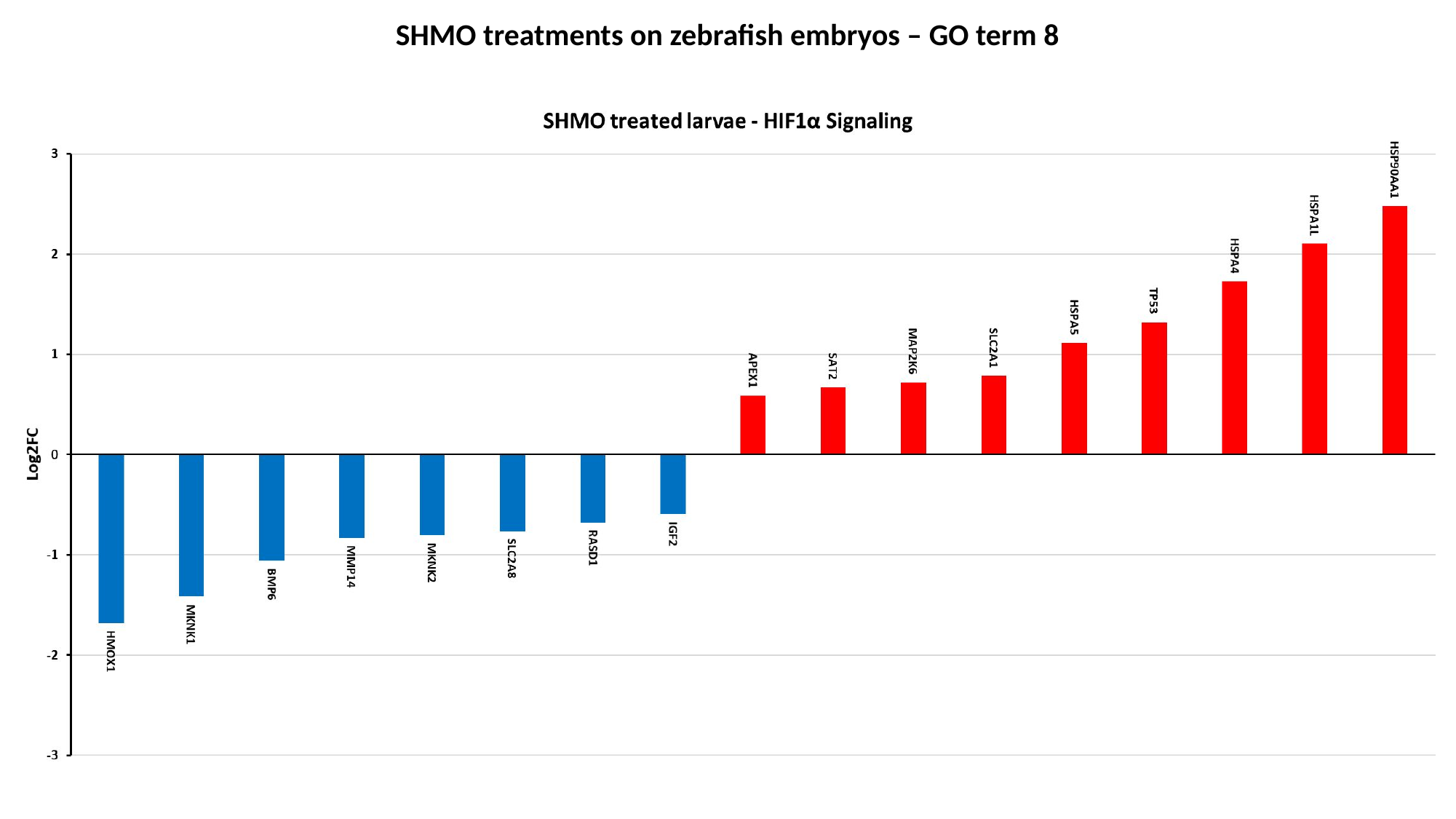

SHMO treatments on zebrafish embryos – GO term 8

## Slide 10
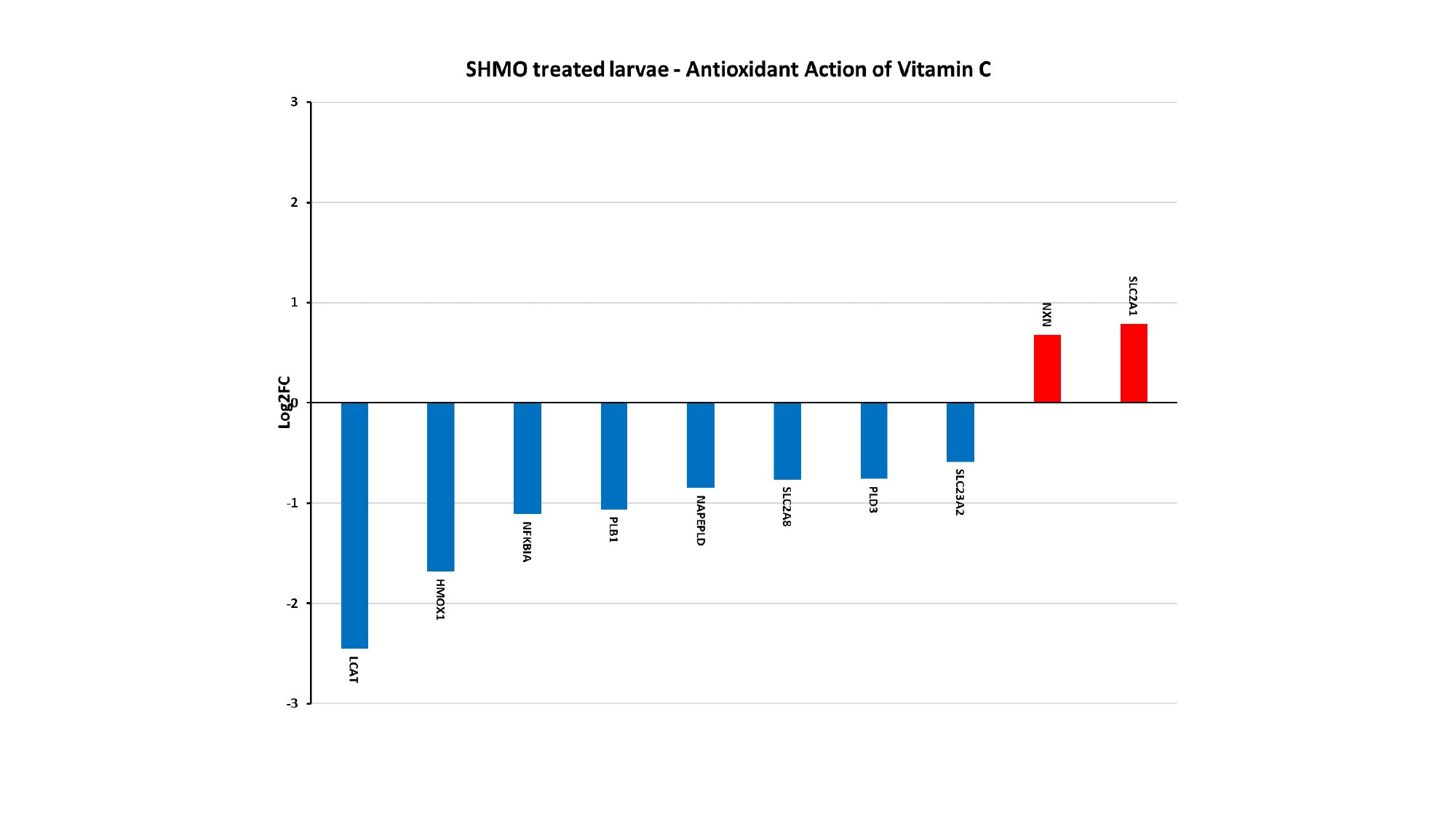

## Slide 11
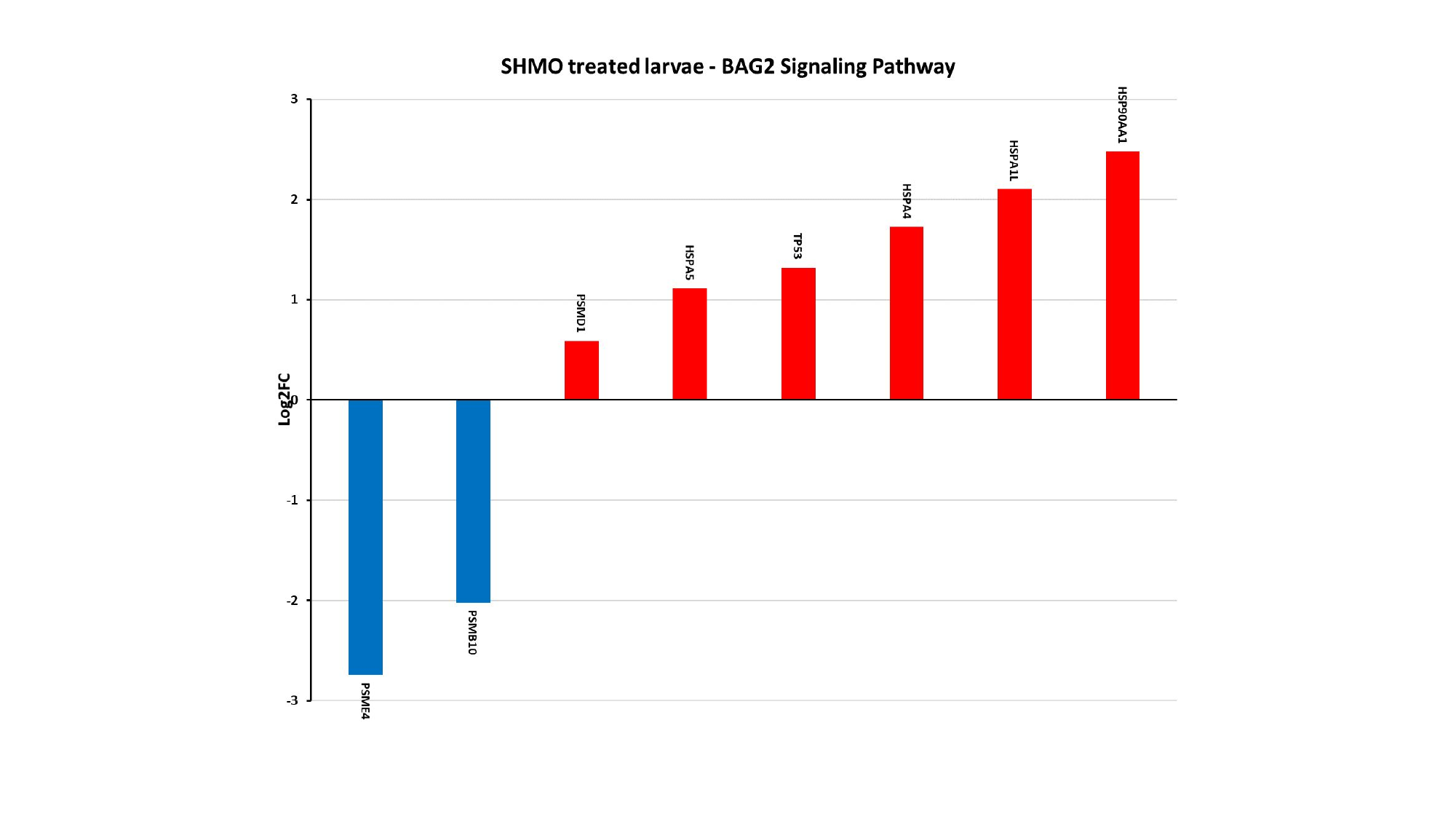

## Slide 12
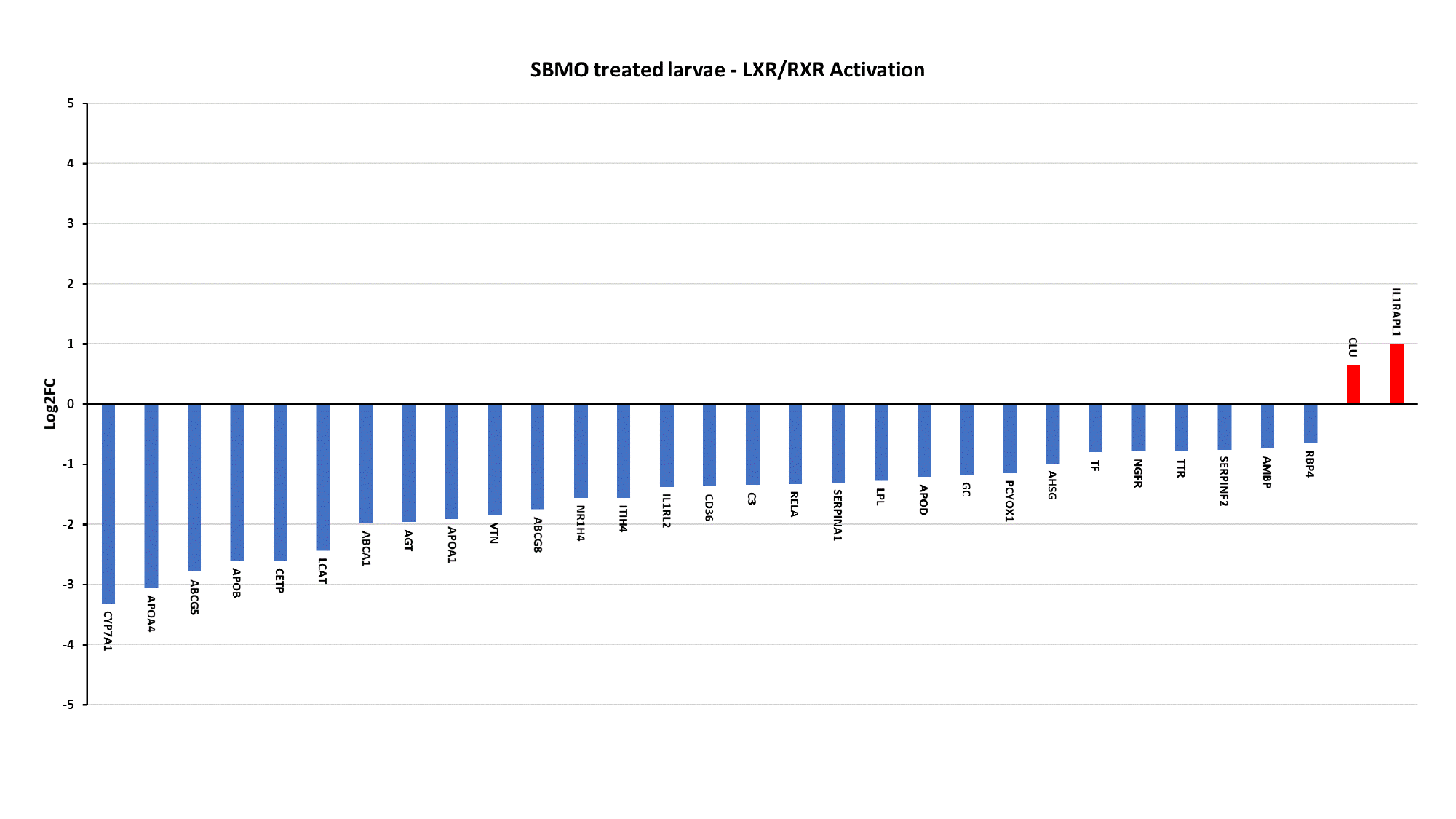

## Slide 13
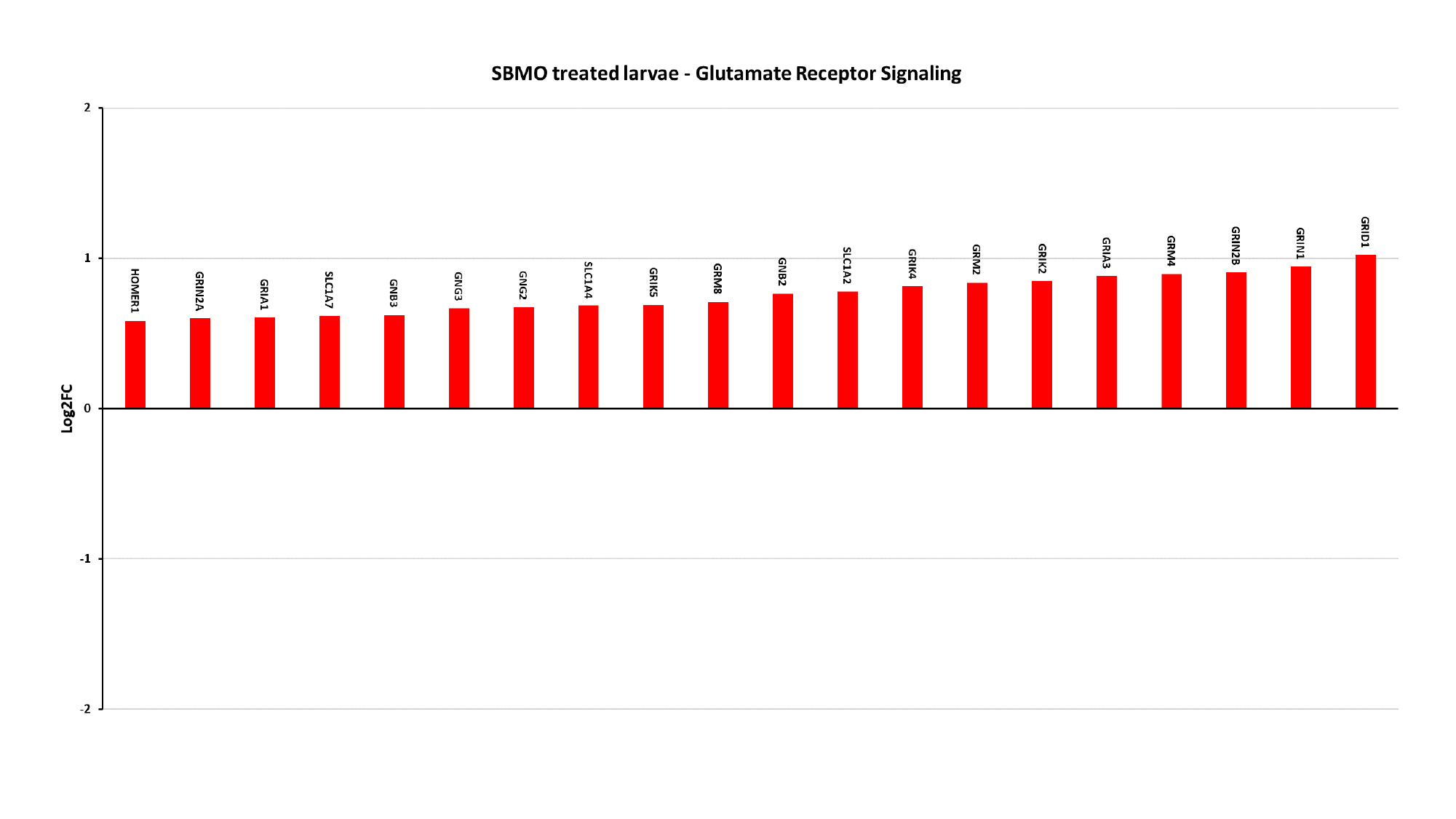

## Slide 14
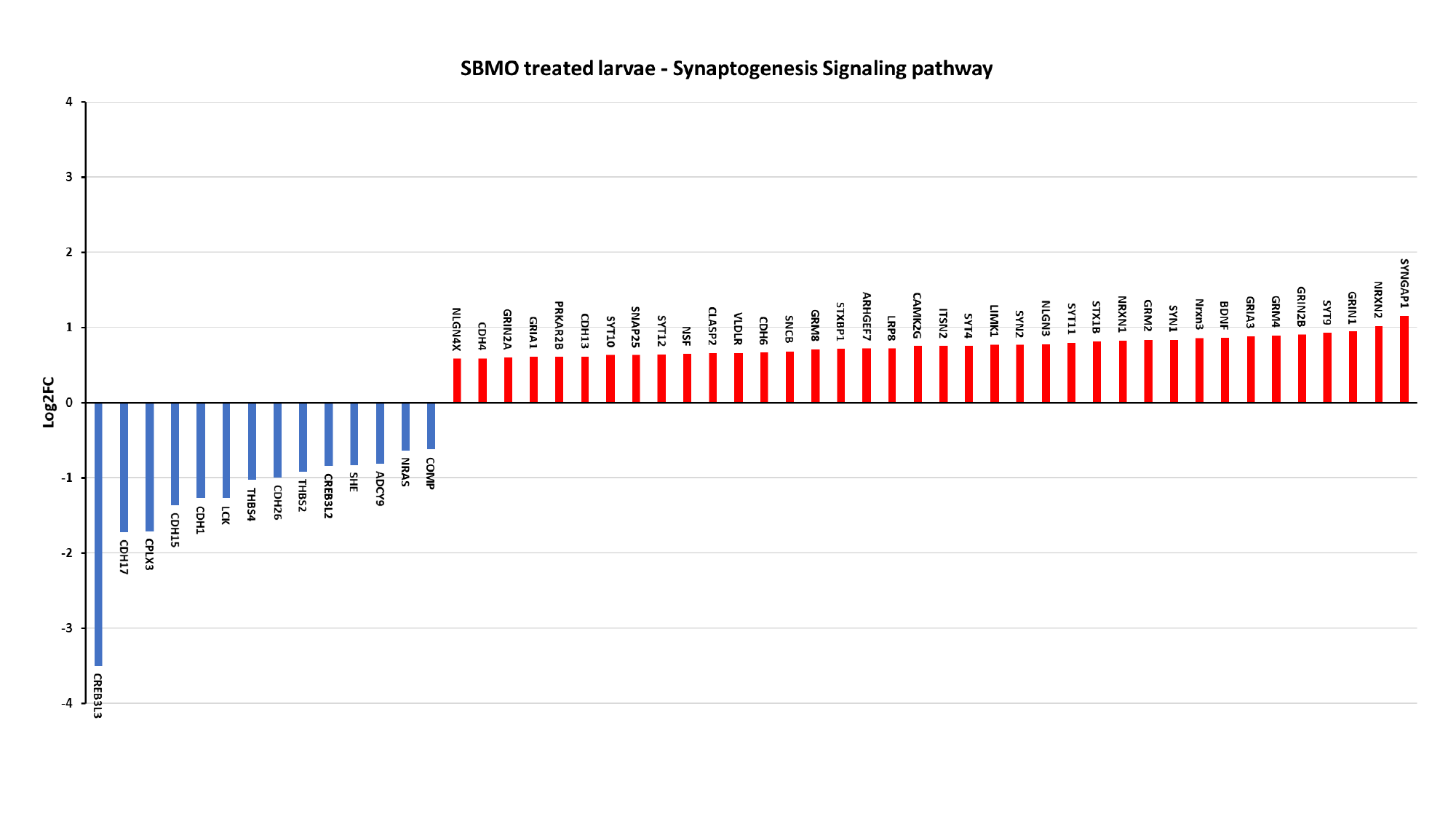

## Slide 15
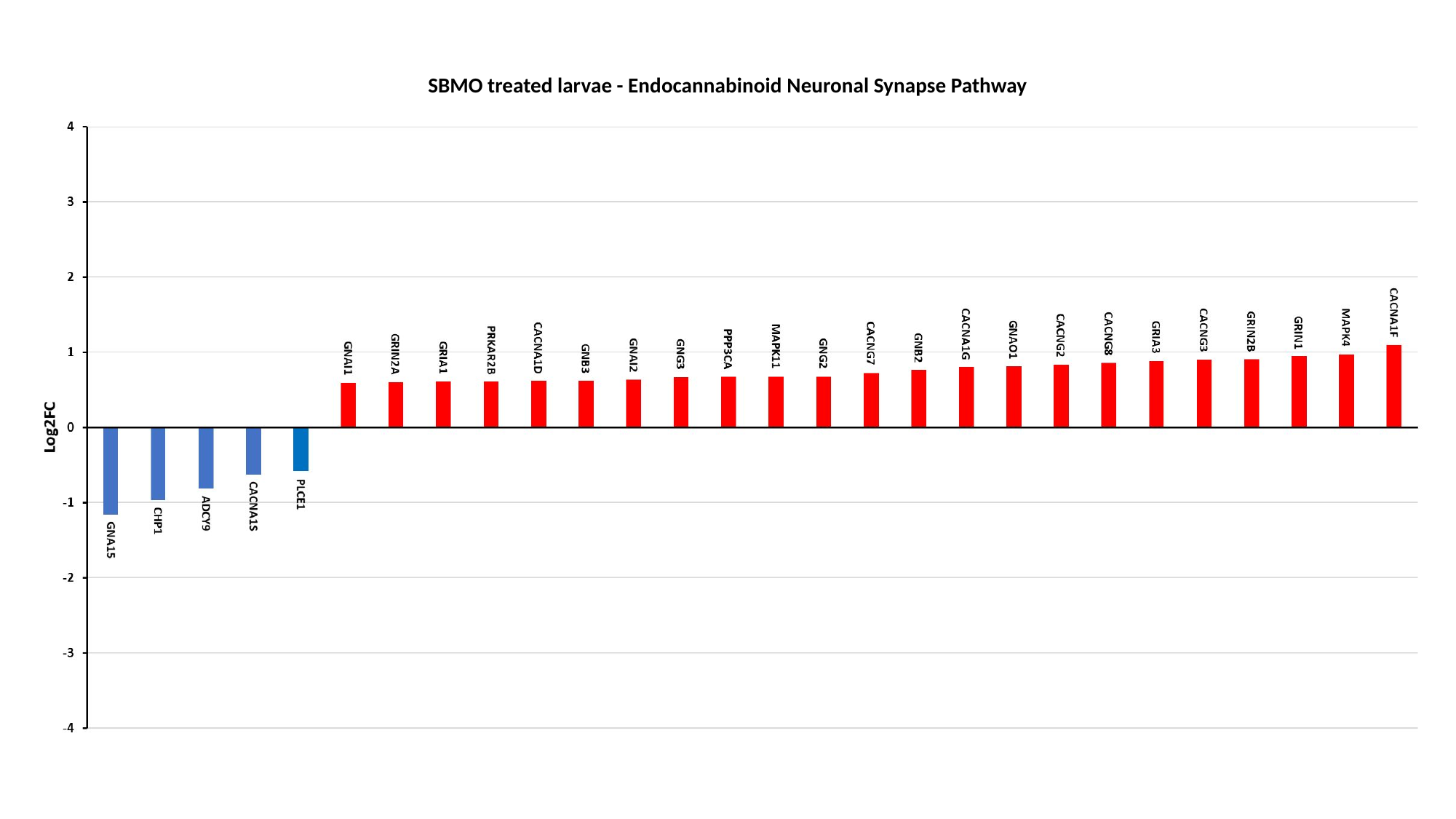

SBMO treated larvae - Endocannabinoid Neuronal Synapse Pathway

## Slide 16
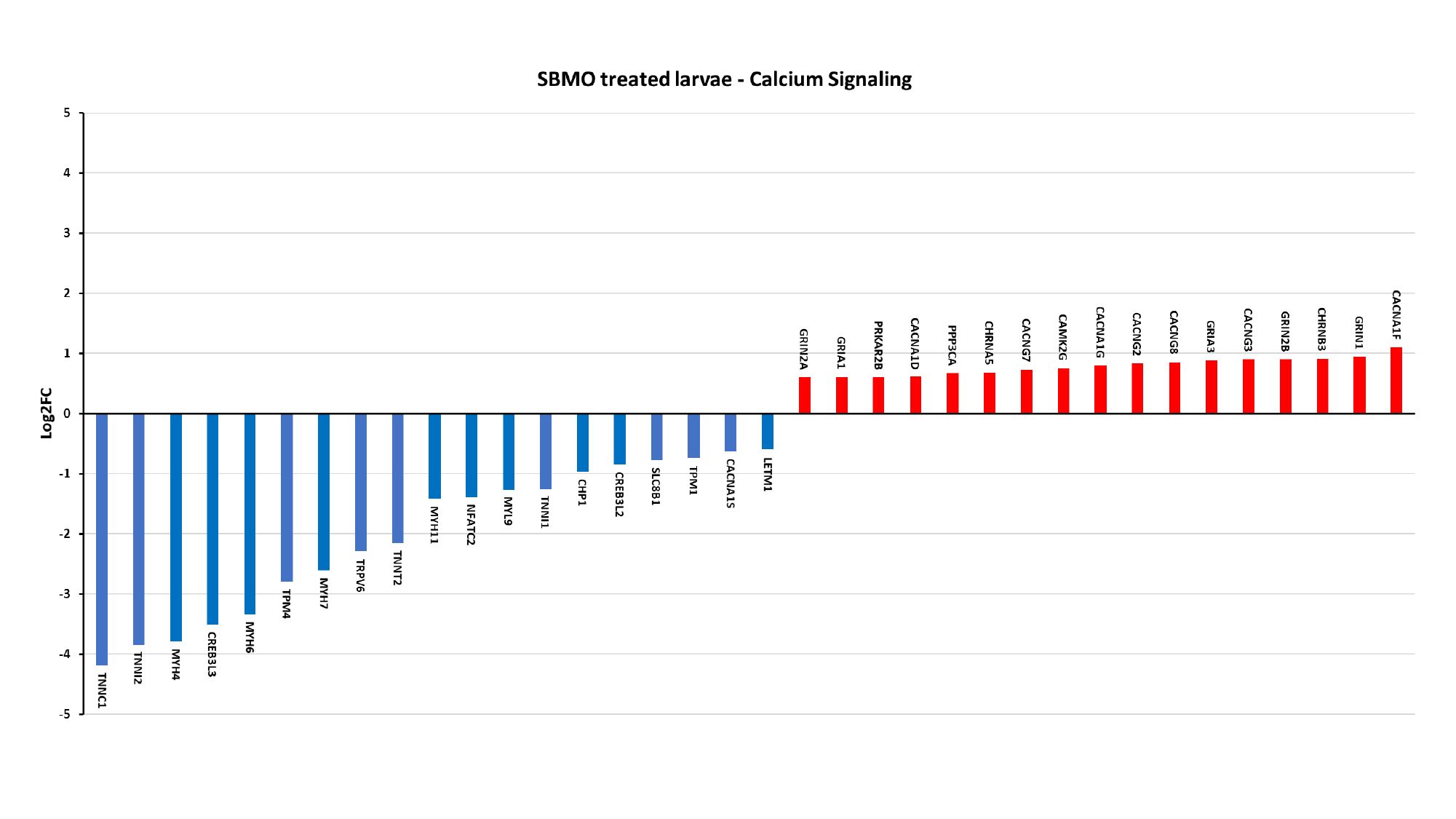

## Slide 17
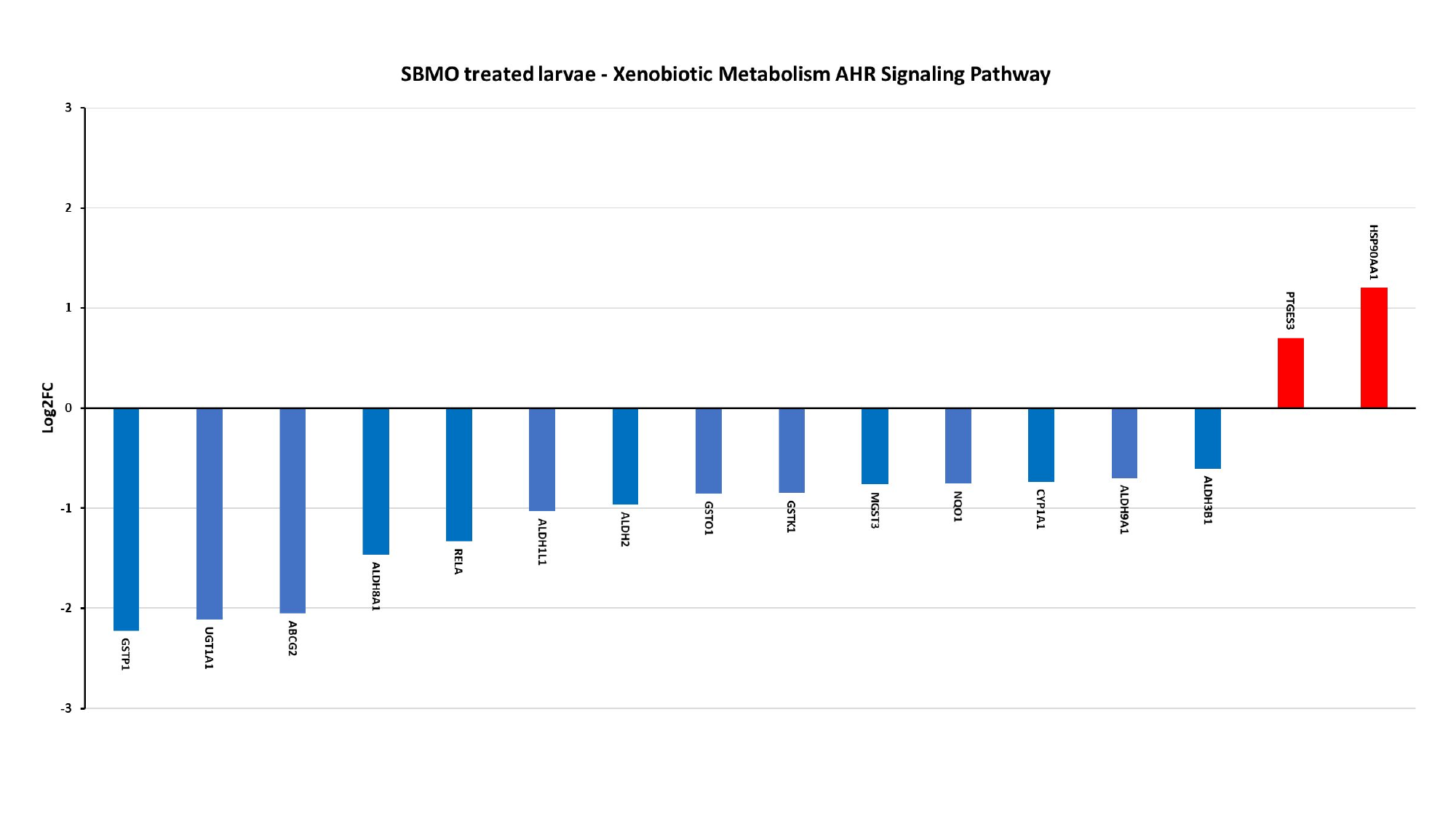

## Slide 18
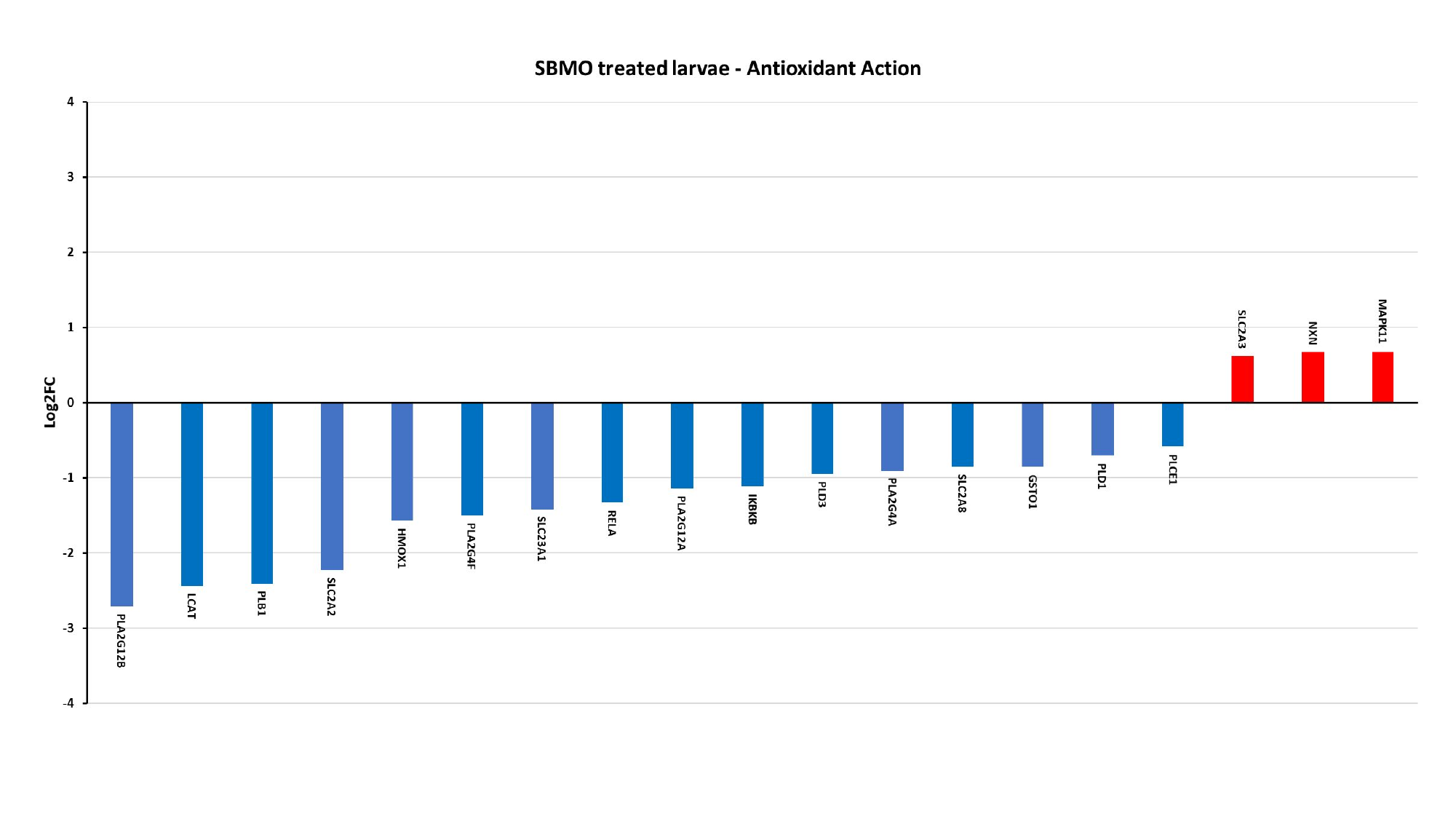

## Slide 19
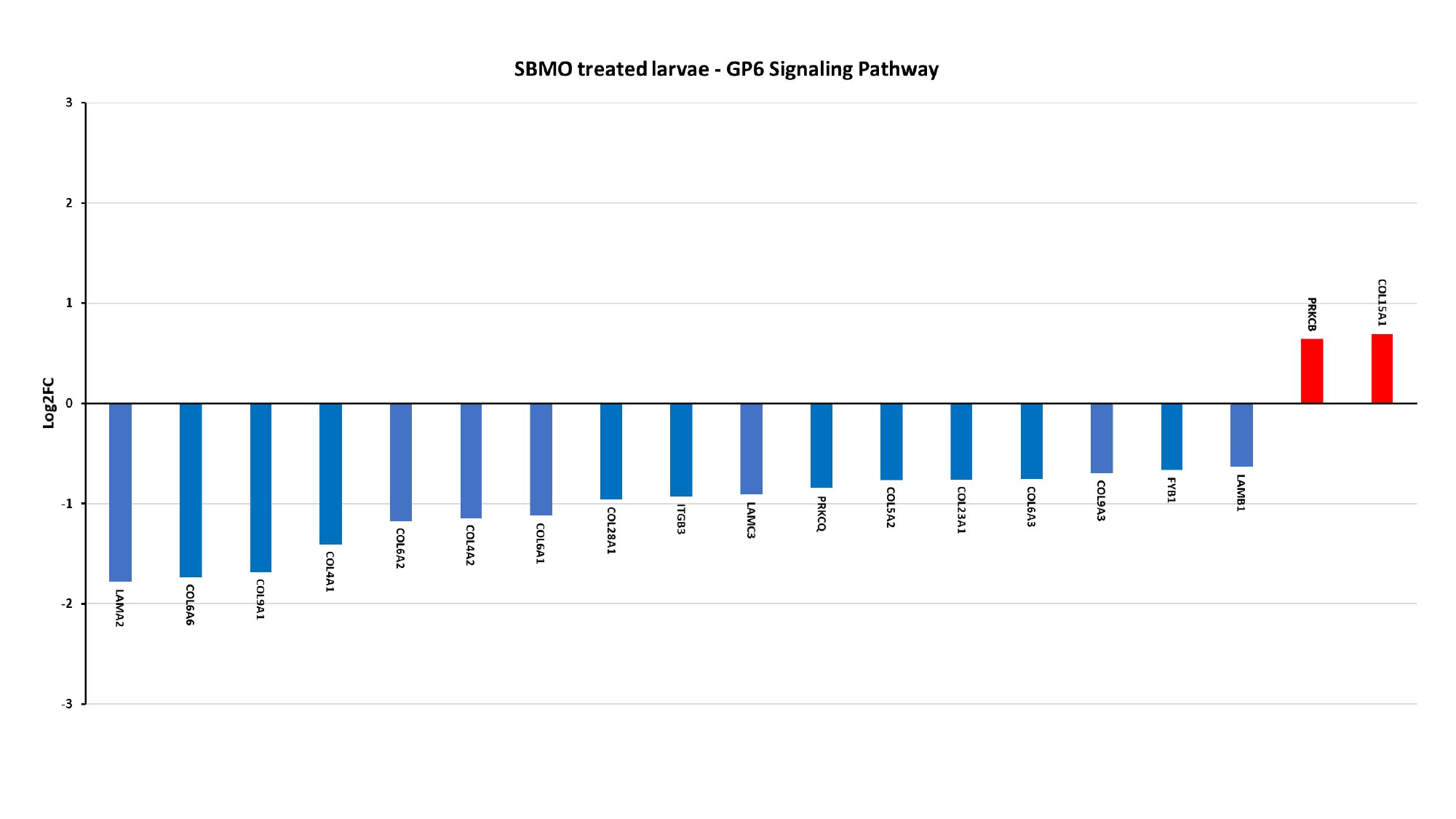

## Slide 20
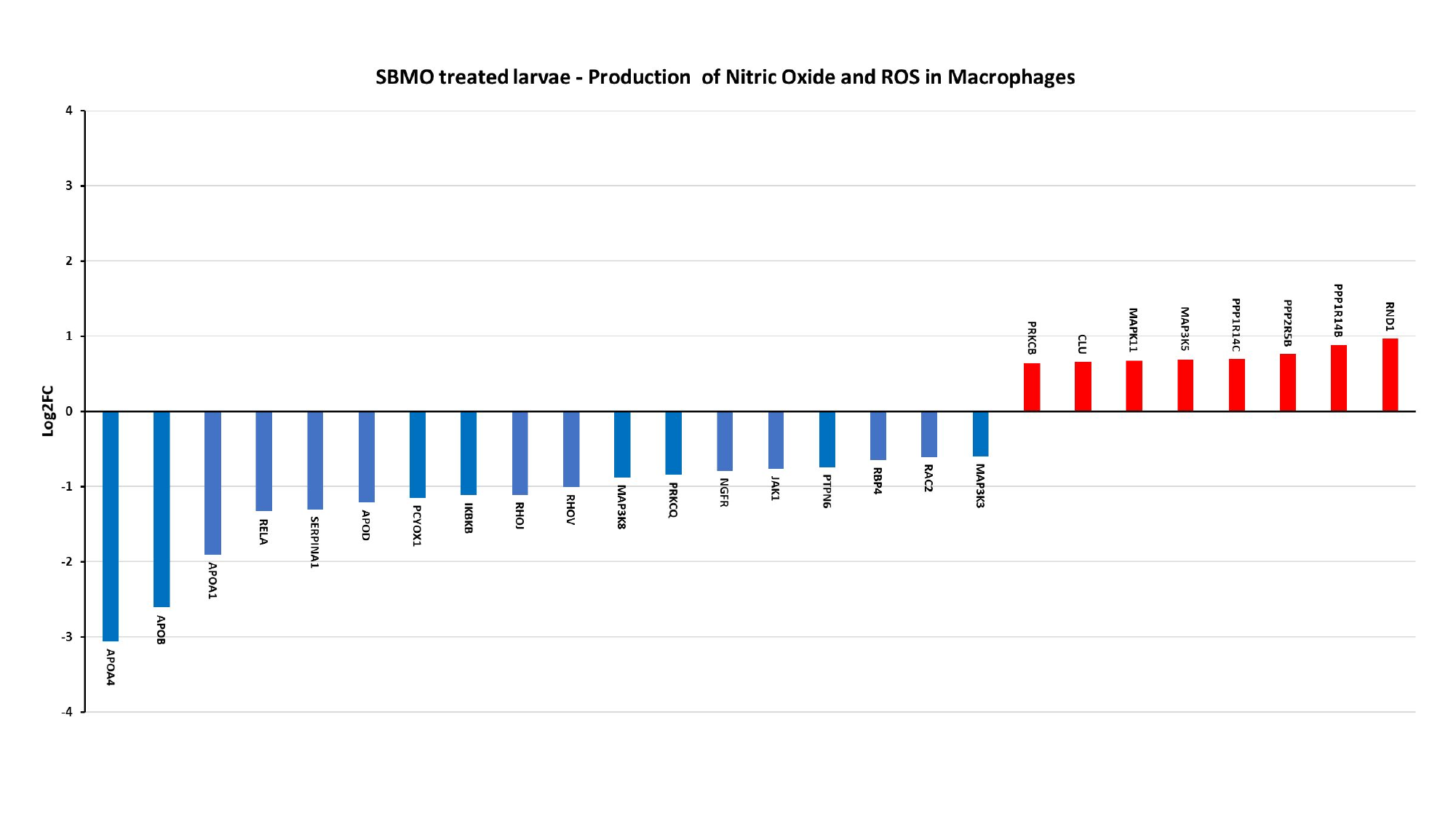

## Slide 21
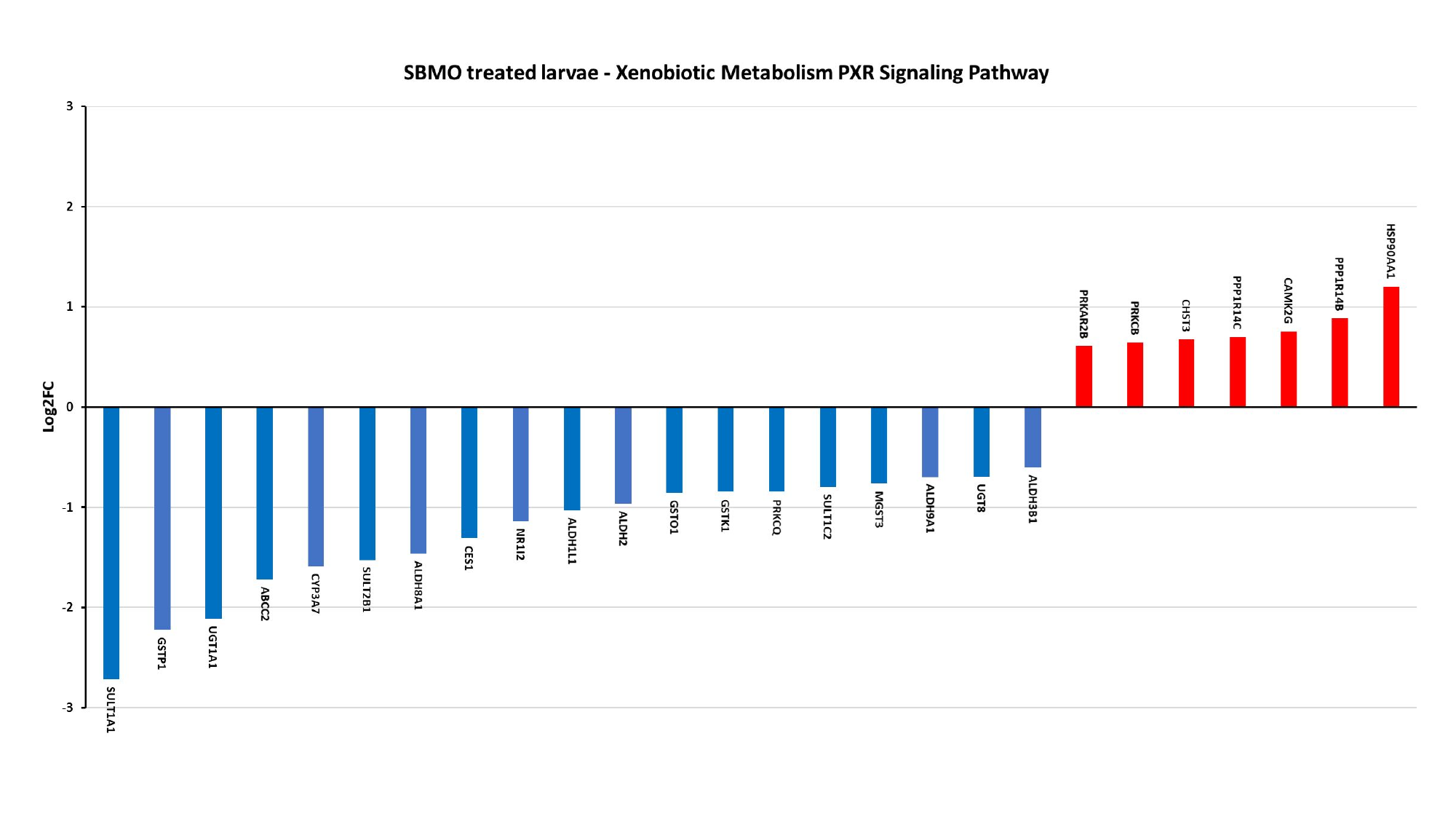

Supplement: Supplementary file 1 [file ijms-24-05456-s001.zip › Licitra et al. - Supplementary Figures.pptx]
